# Western Diet Consumption During Development: Setting the Stage for Neurocognitive Dysfunction

**DOI:** 10.3389/fnins.2021.632312

**Published:** 2021-02-10

**Authors:** Linda Tsan, Léa Décarie-Spain, Emily E. Noble, Scott E. Kanoski

**Affiliations:** ^1^Neuroscience Graduate Program, University of Southern California, Los Angeles, CA, United States; ^2^Department of Biological Sciences, Human and Evolutionary Biology Section, University of Southern California, Los Angeles, CA, United States; ^3^Department of Foods and Nutrition, University of Georgia, Athens, GA, United States

**Keywords:** obesity, high fat diet, adolescent, anxiety, cognition, memory

## Abstract

The dietary pattern in industrialized countries has changed substantially over the past century due to technological advances in agriculture, food processing, storage, marketing, and distribution practices. The availability of highly palatable, calorically dense foods that are shelf-stable has facilitated a food environment where overconsumption of foods that have a high percentage of calories derived from fat (particularly saturated fat) and sugar is extremely common in modern Westernized societies. In addition to being a predictor of obesity and metabolic dysfunction, consumption of a Western diet (WD) is related to poorer cognitive performance across the lifespan. In particular, WD consumption during critical early life stages of development has negative consequences on various cognitive abilities later in adulthood. This review highlights rodent model research identifying dietary, metabolic, and neurobiological mechanisms linking consumption of a WD during early life periods of development (gestation, lactation, juvenile and adolescence) with behavioral impairments in multiple cognitive domains, including anxiety-like behavior, learning and memory function, reward-motivated behavior, and social behavior. The literature supports a model in which early life WD consumption leads to long-lasting neurocognitive impairments that are largely dissociable from WD effects on obesity and metabolic dysfunction.

## Introduction

Children in the United States are exposed to a dietary environment where there is an overabundance of highly palatable foods that are easily affordable and readily accessible. Observations from earlier National Health and Nutrition Examination Surveys (2003–2004, 2005–2006) report that the highest sources of energy for 2- to 18-year-olds were grain desserts, pizza, and soda, which are low in beneficial nutrients, but high in solid fats and/or added sugars (Reedy and Krebs-Smith, 2010). More recent data indicates that consumption of saturated fat and sugar in children continues to exceed the recommended limit of fewer than 10% of total calories for anyone 2-years-old or older, as boys and girls (age 1–18) obtain a range of about 11–12% of their total calories on average from saturated fat and a range of about 11–17% of their total calories on average from added sugar (health.gov, 2015). This type of dietary environment, along with a shift towards larger food portions, has undoubtedly contributed to the alarming increased prevalence of childhood obesity, which is now approximately 18% in children aged 2–19 years (CDC, 2019). In addition, the majority of children with obesity remain obese, both as adolescents and as adults (Simmonds et al., 2016). Emerging evidence reveals that both childhood and adult obesity are associated with impaired performance in various cognitive tasks (Morris et al., 2015; Wang et al., 2016; Dye et al., 2017). However, given that obesity is strongly associated with consumption of a Western Diet (WD; specified in more detail below), a standing question arises as to whether the WD *per se* may impart neurocognitive dysfunction independent of obesity and/or its associated metabolic impairments. Indeed, evidence from both humans and preclinical rodent models described herein indicates that habitual consumption of a WD during early life developmental periods can lead to long-lasting neurocognitive dysfunction even independent of obesity and severe metabolic dysfunction (Francis and Stevenson, 2013; Noble and Kanoski, 2016). Thus, in order to better inform policies relating to dietary recommendations, it is imperative to understand the dietary and neurobiological mechanisms linking perinatal and childhood WD consumption with impaired cognitive abilities throughout the lifespan.

To study the link between WD patterns during early life periods and neurocognitive development, rodent models are often used to target discrete periods of development during which dietary components can be administered with rigorous control and with objective quantification of the amount of calories consumed. The perinatal period in rodents, lasting from gestation to weaning (weaning at ∼postnatal day [PN] 21–24), is a time during which maternal exposures can have lasting effects on cognitive processes. Indeed, the perinatal developmental stage is a critical period for neuronal programming of regions involved in learning and memory, such as the medial prefrontal cortex (mPFC) and the hippocampus (HPC) (Reynolds et al., 2017; Sharp and Lawlor, 2019). Although the exact timing varies slightly by strain, in rats PN 22–27 is considered the approximate juvenile stage of development, PN 28–42 equivalent to the early-mid adolescent period (∼12–17 years in humans), and PN 43–55 comparable to the late adolescence/emerging adulthood period in humans (∼18–25 years) (Spear, 2016). The juvenile and adolescent phases of development are critical periods through which complex cognitive abilities such as working memory, sociability, and inhibitory control develop (Semple et al., 2013).

In laboratory rodents, several different dietary manipulations have been used to model aspects of the WD. A rodent “high fat diet” model typically involves increasing the amount of fat (as a % of total kcal, e.g., 45% or 60% kcal from fat) while reducing the amount of carbohydrates compared to low fat and high carbohydrate standard rodent control diets. However, the carbohydrate content of a common rodent high fat diet is predominantly comprised of simple sugars, vs. the complex polysaccharide-based carbohydrate content of a typical rodent control diet (with the exception of some low-fat control diets that are high in sucrose). Another common rodent WD model is a cafeteria diet, which is a free-choice diet with combination of highly palatable, energy dense foods (e.g., high saturated fat, high sugar) that are commonly consumed by humans. Modeling the obesogenic environment omnipresent in modern Westernized cultures, these diets are provided in the home cages and are therefore easily accessible to the animals. Relative to a control group on a healthy diet, rodents exposed to these WD models may, but do not always, display one or more of the following outcomes associated with metabolic syndrome and obesity: increased caloric intake, body weight gain, increased adiposity, hyperinsulinemia, hyperglycemia, glucose intolerance, and inflammation contributing to hepatosteatosis (Sampey et al., 2011).

While commonly referred to as “diet-induced obesity” (DIO) models in the literature, rodent WD models lead to differential metabolic and body weight outcomes based on species (e.g., rats vs. mice), strain, sex, and/or age and duration of dietary consumption. For example, the same dietary manipulation may lead to obesity and metabolic dysfunction in adult male rats, but not in adolescent female rats. Thus, in this review we will refer to these dietary models as “WDs” and not DIO, and we further define a WD as a rodent model with access to either a diet high in fat (greater than 30% of total kcal from fat), a diet with carbohydrate consumption coming predominantly from monosaccharides (glucose, fructose) or disaccharides (sucrose), or more commonly, a combination of the two (as in the examples described in the previous paragraph).

Herein we review insights from carefully controlled rodent studies that inform on the impact of WD consumed during early life developmental stages on various cognitive domains, including anxiety-like behavior, learning and memory function, reward-motivated behavior, and social behavior (Supplementary Table 1). A second overarching goal of the review is to describe potential underlying neurobiological mechanisms linking dietary models with cognitive outcomes, and thus we focus exclusively on preclinical rodent models as these models offer a distinct advantage in this regard. Perinatal exposure will refer to maternal WD consumption during gestation, lactation, or both, with additional focus on prenatal exposure in Section 5. Given that the overwhelming majority of rodent model studies do not distinguish between the juvenile and adolescent stages, we use the term “adolescence” to refer to the developmental period from weaning until early adulthood, which in rodents is approximately postnatal (PN) days 21–60. We also describe neurocognitive results (behavioral, molecular) with regards to whether or not these outcomes were accompanied by obesity, thus leading to a concluding framework in which early life WD effects on cognition are largely dissociable from effects on body weight and metabolism.

## Anxiety-Like Behavior

### Behavioral Models and Neural Substrates

While anxiety can be an adaptive emotional response to stressful situations, excessive and/or chronic anxiety can have detrimental health effects in humans and manifest as a clinical anxiety disorder (Fox and Kalin, 2014; Sharp et al., 2015; Juruena et al., 2020). In humans, a number of lifestyle factors are associated with anxiety, including diet. For instance, consumption of added sugars and saturated fat is associated with higher anxiety levels (Masana et al., 2019; Fatemi et al., 2020), and evidence from carefully controlled rodent experiments suggests a causal relationship between diet and anxiety-like behavior. In rodents, anxiety-like behavior is assessed via measurable behavioral changes, including measures of exploration, hypoactivity, suppressed consumption of novel foods (neophagia), and fear-associated freezing behavior. One common assessment of anxiety-like behavior is the open field (OF) test, where increased thigmotaxis, or time spent by the walls of an enclosed apparatus, is indicative of increased anxiety-like behavior. Other common anxiety-like behavioral tests are the elevated plus maze (EPM) procedure, and the conceptually similar elevated zero maze (EZM). Each test is rooted in the positive drive for rodents to explore novel environments, as well as the drive to avoid exposed areas without walls or enclosures. Spending more time in the enclosed arms while making relatively few and infrequent crossings to the open arms of the EPM or EZM is indicative of anxiety-like behavior. Other common assessments of anxiety-like behavior include the novelty suppressed feeding (NSF) task, which measures a rodent’s aversion to eating in a novel environment, the social interaction test, where decreased time spent engaging in social behavior is indicative of anxiety-like behavior, and the light-dark transition task, where a decrease in the willingness to explore the illuminated, unprotected area of the apparatus is suggestive of anxiety-like behavior (Bailey and Crawley, 2009). Finally, measuring corticosterone plasma levels following a stressor (e.g., restraint stress) provides a read-out of hypothalamic-pituitary-adrenal (HPA) axis reactivity, which tends to be heightened in anxiety (Packard et al., 2016).

A dietary influence on anxiety may be indicative of lasting changes to brain structures involved in anxiety-like behavior. Briefly, anxiety-like behavior is mediated by a network of brain regions (Adhikari, 2014; Calhoon and Tye, 2015) that is still incompletely understood, but includes the basolateral amygdala (BLA) (Singewald et al., 2003; Hale et al., 2008), the ventral HPC (Nascimento Häckl and Carobrez, 2007; Lowry and Hale, 2010), and the mPFC (infralimbic and prelimbic areas) (Kim et al., 2011; Jiao et al., 2015; Liu et al., 2020). In particular, BLA inputs to the ventral HPC is associated with anxiolytic behavior (Pi et al., 2020) whereas inputs from the ventral HPC to the lateral hypothalamic area (LHA) has been shown to mediate anxiogenic behavior (Jimenez et al., 2018). Additionally, excitation of the BLA terminals in the central amygdala (CeA) is associated with anxiolytic behavior (Tye et al., 2011) whereas CeA projections to the bed nucleus of the stria terminalis are associated with anxiogenic behavior (Ahrens et al., 2018). Finally, brainstem regions are also involved in the control of anxiety-like behavior, with the locus coeruleus being associated with anxiogenic behavior (Itoi and Sugimoto, 2010; McCall et al., 2017). The following sections describe several rodent studies that investigate both the impact of WD on anxiety-like behavior and putative underlying neurobiological mechanisms. Interestingly, several studies discussed below reveal that WD consumption affects similar brain regions in rodents as previously described to be associated with anxiety in humans (Bas-Hoogendam et al., 2017; Besteher et al., 2017, 2020).

### Perinatal WD Exposure

Studies on the effects of perinatal exposure to WD suggest increased anxiety-like behavior in offspring (Bilbo and Tsang, 2010; Peleg-Raibstein et al., 2012; Sasaki et al., 2013; Glendining et al., 2018; Guedine et al., 2018; Winther et al., 2018). For example, when rodent dams are fed a high dietary fat composition (60% kcals fat) before mating until weaning of the offspring, the male (Bilbo and Tsang, 2010; Peleg-Raibstein et al., 2012; Sasaki et al., 2013; Winther et al., 2018) and female (Peleg-Raibstein et al., 2012; Sasaki et al., 2013; Winther et al., 2018) progeny as adults display increased anxiety-like behavior in the EPM apparatus and in a food neophobia task (Peleg-Raibstein et al., 2012) relative to progeny born to dams that received a low-fat diet. In mice, perinatal exposure to diet with a lower fat content (45% kcals fat) also resulted in greater anxiety-like behavior in the EPM at adulthood, especially in females (Glendining et al., 2018). Similarly, male and female offspring from dams consuming a 60% fructose diet presented an anxiety phenotype in the EZM when tested during early adolescence (PN 26–34) (Bukhari et al., 2018). Interestingly, these findings can occur independent of the potential obesogenic effects of the maternal diet on the offspring, as weight gain was not observed in adulthood in one study that found increased anxiety-like behavior in the OF and EPM tasks in adult male and female offspring after perinatal exposure to a 60% kcals fat diet (Sasaki et al., 2013).

While an obesogenic phenotype is not necessary for the development of anxiety-like behavior, the duration of the maternal diet may be an important factor. For instance, if a cafeteria diet is provided to rat dams strictly during lactation only (PN 1–21), the male offspring do not display anxiety-like behavior in the EPM during adulthood (Guedine et al., 2018). This occurs despite hyperphagia and significant weight gain and greater adiposity in the offspring, thus further supporting the notion that while obesity is not a requirement for effects of perinatal WD on anxiety-like behavior, the diet duration from gestation to lactation is critical. In some cases, maternal consumption of a cafeteria diet only during lactation has an anxiolytic impact (decreasing anxiety) on male and female offspring behavior at weaning (Speight et al., 2017) or 10 weeks of age (Wright et al., 2011). The anxiolytic effects associated with maternal cafeteria diet during lactation only may be based on maternal behavior, as Speight and colleagues (Speight et al., 2017) observed enhanced licking and grooming of pups by WD-fed dams.

In addition to the effect of perinatal WD exposure on anxiety-like behavior, studies suggest that maternal WD consumption throughout gestation and lactation may impact the HPC and amygdala, brain regions that are strongly linked with anxiety. For example, WD-associated anxiety-like behavior is accompanied by increased expression of 5HT-r1a and GABAa alpha2 receptor subunit in the ventral HPC (Bannerman et al., 2004), as well as elevated brain-derived neurotrophic factor (BDNF) expression in the dorsal HPC (Peleg-Raibstein et al., 2012), a region where BDNF levels correlate with the magnitude of anxiety-like behavior in the EPM task in wildtype mice (Yee et al., 2007). Exploration of the open arm of the EPM apparatus (decreased by WD) also correlates negatively with HPC gene expression for inflammatory markers TNFa and MCP-1 (Winther et al., 2018). Pups perinatally exposed to WD have elevated hippocampal microglial activation at birth, as demonstrated by increased expression of CD11b, a microglial activation marker, and TLR4, an endogenous pattern recognition receptor involved in metabolic-inflammatory signaling (Bilbo and Tsang, 2010). Additionally, pups exposed to a perinatal WD also show increased circulating peripheral cytokine expression (IL-1β in the liver and serum IL-6) during adulthood (Bilbo and Tsang, 2010). In the amygdala, perinatal WD elevates mineralocorticoid and glucocorticoid receptors during adulthood in rats, possibly due to an overall heightened HPA axis response to stress supported by the elevated basal corticosterone levels also seen in the adult rats (Sasaki et al., 2013). Taken together these findings suggest that perinatal exposure to a WD promotes inflammatory processes and alters stress responsivity markers. Indeed, others have found that maternal obesity is associated with increased inflammatory signaling during pregnancy that likely impacts the development and health of the offspring (Segovia et al., 2017), and that the HPA axis during development is vulnerable to maternal nutrition and/or metabolic status (Long et al., 2012; Balsevich et al., 2016). Collectively, these findings suggest that maternal WD consumption impacts the brain in a multitude of ways that may impact anxiety-like behavior, including increasing inflammatory signaling pathways, modification of the serotonergic, GABAergic, and neurotrophin signaling systems, and elevating the HPA axis responsiveness. Further research is necessary to determine the extent to which these neurobiological changes are causally related to the impact of perinatal WD exposure on anxiety-like behavior.

While the majority of the studies described above found anxiogenic effects (increased anxiety) as a consequence of perinatal WD exposure, some have reported mixed results in various anxiety measures (Sasaki et al., 2014; Zieba et al., 2019). For example, perinatal exposure to a 60% fat diet reduced anxiety-like behavior in the light-dark box while having anxiogenic effects in the EPM and OF tasks (Sasaki et al., 2014). Similarly, although anxiety measures in the OF and NSF tests were unchanged by maternal consumption of a WD, a trend for increased open arm time in the EPM was observed at adulthood (Zieba et al., 2019). Such findings raise the question as whether certain behavioral assays are more sensitive to the anxiogenic impact of perinatal WD consumption.

In contrast to the anxiogenic effects associated with perinatal WD exposure described above, maternal exposure to a WD may be anxiolytic (decreasing anxiety) for the offspring in the presence of perinatal stress. For example, rat offspring that underwent maternal separation, which normally induces anxiety, did not display increased anxiety-like behavior in the OF test if the dams consumed a WD (40% kcals fat) from gestation to postpartum day 21 (Rincel et al., 2016). These results were accompanied by a WD-associated prevention of maternal separation-associated changes in the expression of several genes in the PFC that are linked with abnormal anxiety-like behavior in adulthood, including BDNF and 5HT-r1a (Rincel et al., 2016). Moreover, the maternal separation-induced upregulation of Rest4 in the PFC, whose expression is associated with anxiety in adulthood (Uchida et al., 2010), was reversed with perinatal WD (Rincel et al., 2016). These data suggest a potential relationship between perinatal WD and stress on anxiety-like behavior later in life.

### Adolescent WD Consumption

Similar to perinatal exposure, adolescent consumption of WD can lead to increased anxiety-like behavior. For example, adolescent male rats that consumed a cafeteria diet consisting of 45% fat, a 15% weight by volume (w/v) sucrose solution, and standard chow during adolescence displayed increased anxiety-like behavior in the EPM during adulthood (Ferreira et al., 2018). Similarly, consumption of a 45% fat diet combined with a 10% w/v sucrose solution for 8 weeks promoted anxiety-like behavior in adulthood (Gancheva et al., 2017). In male mice, 7 weeks of exposure to a 60% fat diet enhanced anxiety-like behavior in both the EPM and the OFT (Yang et al., 2020). Male, but not female, adolescent rats given free access to chocolate cookies (high in both fat and sugar) presented an elevated anxiety phenotype in the EPM as well as greater plasma corticosterone levels following restraint stress (Kim et al., 2018). Similarly, enhanced HPA axis reactivity was also observed in male rats fed a lard-based high fat diet (60% kcals fat) from adolescence through adulthood, although no diet effects were reported in the OF task (Abildgaard et al., 2014). In addition, ad lib access to a 5% w/v sucrose solution in adolescent male rats from PN 30–46 was sufficient to induce an anxiety phenotype in the NSF task months later when tested during adulthood (PN 204) (Gueye et al., 2018), and male rats that consumed a 10% w/v sucrose solution from PN 25–50 also displayed anxiety-like behavior in the OF test in adulthood (PN 75) (Kruse et al., 2019). However, the aforementioned study also showed that the long-term effect of increased anxiety-like behavior was not seen in adult male rats that received the 10% w/v sucrose drink from PN 75–100, highlighting early life as a critical time period during which Western dietary patterns influence anxiety-like behavior (Kruse et al., 2019). Together, these studies support that, similar to perinatal WD consumption, exposure to WD factors during early adolescence generally promotes anxiety-like behavior in adulthood.

Similar to effects associated with perinatal WD, anxiety-like phenotypes are observed independent of weight gain and obesity outcomes caused by adolescent WD consumption. For example, rats that consumed a cafeteria diet consisting of 45% fat, a 15% w/v sucrose solution, and standard chow during adolescence had significantly increased caloric intake and body weight (Ferreira et al., 2018), but consumption of a marginally high fat diet (21.1% from fat) from 1 to 5 months old in male mice did not result in differences in body weight relative to controls (Vinuesa et al., 2016). However, both studies found that these rodents developed anxiety-like behavior in adulthood after consuming the WD. In addition, the increased anxiety-like behavior discussed above in adolescent male rats consuming a lard-based high fat diet (45% kcals fat) with a 10% w/v sucrose solution for 8 weeks was associated with features of the metabolic syndrome such as reduced insulin sensitivity, hypercholesterolemia, hypertriglyceridemia and greater visceral adiposity (Gancheva et al., 2017) despite no differences in body weight, thus implying a potential role for metabolic impairments rather than increased body mass *per se*.

While weight gain may be less relevant to the development of anxiety-like behavior associated with adolescent WD consumption, WD may be contributing to anxiety-like behavior by affecting neurobiological processes in the HPC, the nucleus accumbens (ACB) and the mPFC. For example, Ferreira and colleagues (Ferreira et al., 2018) found that anxiety-like behavior induced by adolescent WD consumption is associated with reduced neurogenesis in the subgranular region of the dentate gyrus. In accordance, cell proliferation was diminished in the dentate gyrus of adult rats exposed to a sucrose solution during adolescence (Gueye et al., 2018). Kim and colleagues (Kim et al., 2018) observed anxiogenic phenotype associated with chocolate cookies consumption that coincided with increased BDNF expression in the ACB, a feature reminiscent of rodent stress-induced depression models (Eisch et al., 2003). Male rats fed a 60% fat diet for 7 weeks displayed greater senescence-related gene expression in the mPFC, especially in astrocytes and microglia (Yang et al., 2020). Furthermore, Kruse and colleagues (Kruse et al., 2019) found that anxiety-like behavior seen in male rats that consumed a 10% w/v sucrose solution during adolescence may be explained, in part, by increased mPFC, but not ventral HPC, expression of calretinin, an important developmental calcium-binding protein that is increased after stressful situations such as maternal separation (Xu et al., 2011) and whose protein expression is usually reduced in adulthood (Caballero et al., 2014). Importantly, differences in calretinin expression were not observed in males that consumed the high sucrose diet during adulthood, suggesting that excessive sucrose consumption during adolescence impacted normal calretinin development. Despite these findings, more research is needed to identify the physiological relevance of each of these candidate pathways to early life WD-induced effects on anxiety-like behavior.

While many studies show that WD consumption during adolescence generally promotes anxiety-like behavior, it should be noted that there are instances where an elevated anxiety phenotype did not develop after consumption of WD. For example, brief exposure (11 days) to a 41% fat diet initiated at adolescence (PN31) had no impact on anxiety-like behavior in the EPM (Vega-Torres et al., 2020). In male mice, prolonged consumption of a 45% fat diet failed to induce behavioral changes in the OF test and the EPM, when testing occurred after 8 and 10 weeks of diet, respectively (Del Rio et al., 2016). Similarly, intake of a 55% fructose diet initiated at adolescence did not alter anxiety measures in the OF and EPM tests, although basal corticosterone plasma levels were increased after 10 weeks on the diet in male rats (Harrell et al., 2015). One study showed that a cafeteria diet resulted in anxiolytic behavior in adulthood in the OF and EPM test in male and female rats when fed from weaning until early adulthood (3–11 weeks old) (Lalanza et al., 2014). Results revealed that the cafeteria diet increased adiposity and metabolic disturbances, such as hypertriglyceridemia, hyperglycemia and insulin resistance, in both males and females. However, a 1-week removal of the cafeteria diet during adulthood led to increased anxiety in the OF test, suggesting that cafeteria diet withdrawal, but not the cafeteria diet itself, can prompt anxiety-like behavior (Lalanza et al., 2014). However, additional studies are needed given that anxiety-like behavior can still be seen in animals that still consume a WD into adulthood (Vinuesa et al., 2016). Other studies have shown that excessive sugar consumption (11% w/v sucrose or high fructose corn syrup solution) during adolescence has no impact on anxiety-like behavior during adulthood in male rats in the EZM (Hsu et al., 2015; Noble et al., 2019). Whether or not anxiety-like behavior is developed after chronic sugar access may depend on the time of testing following sugar removal. For instance, Kruse et al. (2019) found anxiety-like behavior in male rats following 25 days of 10% w/v sucrose removal, whereas Noble et al. (2019) did not find any differences in anxiety-like behavior almost 4 months after removal of an 11% w/v high fructose corn syrup solution. Interestingly, using a similar experimental design to Noble et al., Hsu et al. saw no differences in anxiety like behavior in rats fed either 11% sucrose solution or 11% HFCS solution when testing occurred with no delay following sugar consumption (Hsu et al., 2015). Altogether, these studies suggest that withdrawal from WD may in part explain the increased anxiety-like behavior seen in rodents, although this effect may depend on the type of WD (sugar, high-fat, or combination of the two) and the anxiety-like phenotype may be alleviated given significant time consuming a healthy control diet.

Similar to effects associated with perinatal WD, consumption of WD during adolescence in rodents may reduce anxiety in circumstances associated with early life stress (ELS), fostered by either maternal separation, restraint, social isolation, or a disrupted nest anywhere from PN 2–28. For instance, in male rats, consumption of a high sucrose WD during social isolation from PN 21–28 resulted in reduced anxiety-like behavior in the OF and EPM tests at PN 28 relative to animals that received standard chow and animals that received stress without the WD (Marcolin et al., 2012). Similarly, social anxiety was attenuated in male rats maintained on a WD (45% kcal from fat) after weaning after having previously undergone a 3-day ELS test from PN 27–29 where on each day they were subjected to either forced swim, elevated platform stress, or restraint adulthood (Ali et al., 2018). In male rats subjected to limited nesting from PN 2–9, free access to a high fat/high sucrose diet (43% kcals fat, 17% kcals protein and 40% kcals from sucrose) initiated at weaning also prevented the expression of an anxiety phenotype at adulthood (Maniam et al., 2016). Similarly, female rats subjected to ELS from PN 2–14 also display reduced anxiety, as assessed by EPM in adulthood, following consumption of a continuous cafeteria-style WD (32% kcals from fat) (Maniam and Morris, 2010). Thus, similar to what occurs with perinatal WD, these findings suggest that the relationship between dietary factors and anxiety in adolescents interacts with the effects of ELS. However, this is not always the case, and may be stressor- or age-dependent. For example, exposure to predator stress during adulthood in male rats fed a WD since weaning exacerbated anxiety behaviors in the EPM and OF tests (Kalyan-Masih et al., 2016).

While the underlying mechanisms for the anxiolytic effects of WD consumption in cases of ELS are incompletely understood, WD consumption, either during or following ELS, resulted in rats consuming more food (Marcolin et al., 2012; Maniam et al., 2016), gaining more weight (Maniam and Morris, 2010; Marcolin et al., 2012; Maniam et al., 2016), having increased adipose tissue (Maniam and Morris, 2010; Ali et al., 2018), higher plasma glucose levels (Marcolin et al., 2012), and elevated plasma leptin and insulin (Maniam and Morris, 2010). Normally ELS will result in an elevated corticosterone response, however, consumption of WD following ELS reduces the corticosterone response in adulthood (Maniam and Morris, 2010; Ali et al., 2018). The reduced corticosterone response may be related to the normalized hypothalamic corticosterone releasing hormone mRNA and reduced hippocampal glucocorticoid receptor gene expression seen in adulthood following consumption of a WD in females (Maniam and Morris, 2010), although hippocampal glucocorticoid receptor protein expression was increased in males (Maniam et al., 2016). Moreover, WD may prevent an imbalance of antioxidant enzymes in the PFC (Marcolin et al., 2012) or lead to increased D1R and D2R mRNA expression in the ACB, suggesting that dopamine signaling may also have a role in protecting against stress-induced anxiety-like behavior (Ali et al., 2018). Importantly, these studies suggest that ELS in combination with a WD prevents anxiety-like behavior despite leading to a compromised metabolic phenotype, as demonstrated by weight gain, adiposity, and elevated plasma insulin, leptin, and glucose levels. Collectively, these studies reveal that WD consumption may function as a reaction to stress that can relieve anxiety-like behavior associated with ELS.

### Summary

**FIGURE 1:**
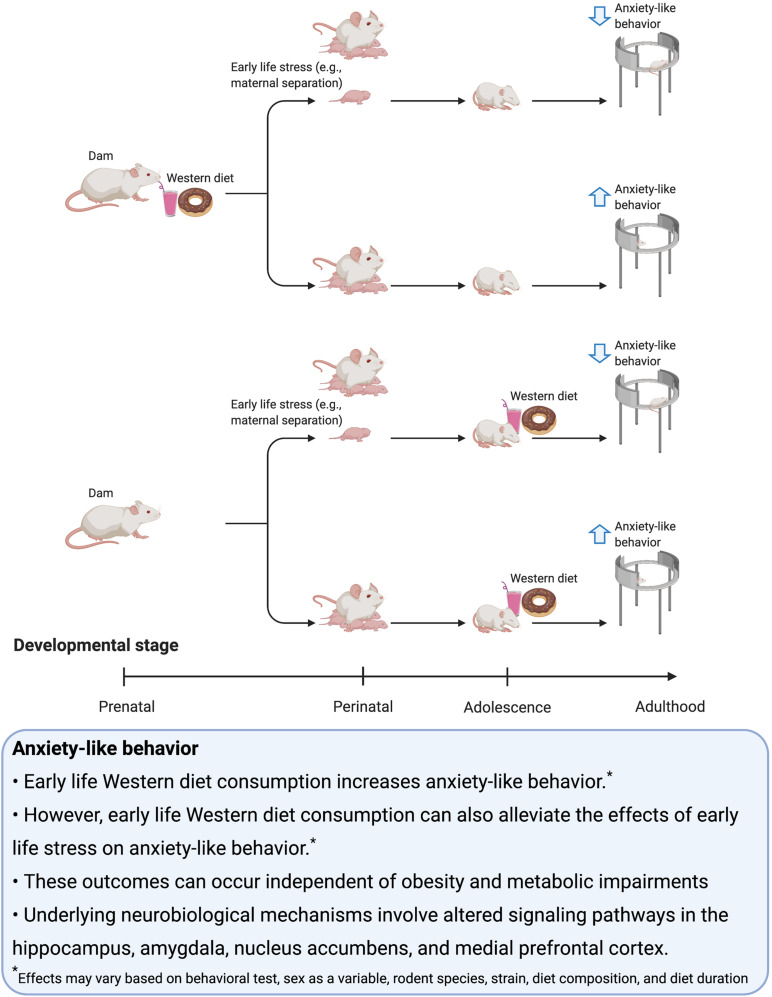
Summary of the effects of early life Western diet consumption on anxiety-like behavior.

The development of anxiogenic or anxiolytic behavior in association with early life WD exposure is likely dependent on whether or not the WD is accompanied by ELS (Figure 1). More specifically, evidence suggests that exposure to WD, either perinatally or during adolescence, is associated with increased anxiety-like behavior during adulthood unless the rodents undergo a period of ELS, in which case the diet may reduce anxiety like behavior. Therefore, WD consumption may be a coping mechanism in response to ELS. Notably, while obesity is often not observed following early life WD consumption, in the cases of ELS, obesity and metabolic dysfunction are often present in those consuming WD factors, thus further highlighting that WD influences on anxiety are not directly tied to the presence vs. absence of obesity and associated comorbidities. Further research is needed to determine the precise mechanisms through which early life WD factors, either with or without ELS, impact the brain and responsivity to stress during adulthood.

## Learning and Memory

### Behavioral Tests

The process of learning and remembering information is perhaps as well-studied as it is persistently mysterious. In rodent models, memory is typically assessed based on an observable behavioral change that is attributable to prior experience, and a variety of behavioral tasks are commonly implemented depending on the type of memory being studied. Common tests of recognition memory that involve passive reinforcement (not inherently appetitive/rewarding, or aversive/punishing) are the Novel Object Recognition (NOR), Novel Place Recognition (NPR), or Novel Object in Context (NOIC). The latter two of these tasks, which involve contextual-based associations, relies on the function of the HPC and interactions with the perirhinal and prefrontal cortices, whereas object recognition memory relies on the function of the perirhinal cortex, and is independent of the HPC when conducted without a temporal or contextual component (Barker and Warburton, 2011).

Spatial memory, or memory based on the location of visuospatial cues in the environment, is commonly assessed via the Y-Maze Spontaneous Alternation task, Morris Water Maze (MWM), the radial arm maze, and the Barnes Maze. These tasks require allocentric navigation, are hippocampal-dependent, and involve either escape from aversive reinforcement (MWM, Barnes Maze), appetitive reinforcement (e.g., food; radial arm maze), or passive reinforcement (Y-Maze Spontaneous Alternation task) (Broadbent et al., 2004; Ofen, 2012; Quillfeldt, 2016). Other memory tests discussed in this section include fear conditioning and avoidance learning (typically based on brief foot shock or predator odor) and Pavlovian stimulus-reinforcement learning. These latter learning and memory tasks are mediated by complex network of forebrain structures that include the amygdala, striatum, insula, HPC, and mPFC among other regions (Kong et al., 2014; Quillfeldt, 2016). WD consumption has been shown to impact the neural substrates associated with the behavioral tasks described above, and early life stages of development are particularly sensitive to these effects in both human and rodent models (Noble and Kanoski, 2016). Moreover, the HPC is a canary in the coal mine in the sense that, at least in some cases, WD factors have a negative influence on hippocampal-dependent memory processes following very short periods of consumption, prior to the effects of the diet on body weight, metabolic function, and integrity in other brain regions (Kanoski and Davidson, 2011). In this section, we discuss the neural substrates and potential mechanisms through which early life WD factors impart learning and memory dysfunction while highlighting some critical gaps in current knowledge.

### Perinatal WD Consumption

Exposure to western dietary factors through maternal nutrition has long-term consequences for learning and memory function, even when the animals are maintained on standard, low-fat chow after weaning (Tozuka et al., 2010; Peleg-Raibstein et al., 2012; Kuang et al., 2014; Page et al., 2014; Lépinay et al., 2015; Bengoetxea et al., 2017; Janthakhin et al., 2017; Vuong et al., 2017; Rincel et al., 2018; Cordner et al., 2019). For example, conditioned odor aversion in which an animal avoids an odor that was previously paired with an aversive memory, is impaired in adult male rats that received perinatal exposure to a WD containing 45% kcal from fat (Janthakhin et al., 2017). Moreover, the ability to extinguish an avoidance response to a previously paired odor is impaired in rats that receive the same perinatal WD exposure for the same duration (Rincel et al., 2018). Similarly, memory in the NOR task is also impaired in both adolescent male and female rats (Moreton et al., 2019) and adult male rats (Teixeira et al., 2020) when their dams consumed a cafeteria diet throughout lactation. Male offspring from dams fed a 45% kcal fat diet during both gestation and lactation failed to discriminate a novel object in NOR at 14 weeks of age as well (Vuong et al., 2017). Interestingly, male progeny from dams fed a 45% kcal fat diet (GD14 – PN21) demonstrated impairments in the NOR task at PN19–20 but not 1–2 months of age in both sexes (Bengoetxea et al., 2017). Together, these studies suggest that object-based episodic memory and the ability to learn and extinguish conditioned odor aversion is weakened with perinatal WD exposure.

In contrast to conditioned odor aversion and NOR, other types of memory may not be as vulnerable to perinatal WD consumption. Spatial memory retention in the MWM task, for example, is actually enhanced in male rats after perinatal exposure to a 60% kcals high-saturated-fat or high-trans-fat diet (Bilbo and Tsang, 2010). In support of this, another study has shown that MWM spatial memory retention was only impaired in adulthood when obese male rats were reintroduced to a WD (60% kcals from fat) later in life at 8 weeks old, despite having received perinatal exposure to the same WD during gestation and lactation (White et al., 2009). Furthermore, while spatial learning in the Barnes Maze was impaired in adolescent male offspring of mouse dams that received a 57% kcals fat WD from 6 weeks prior to mating up to lactation day 16, there were no differences in spatial memory/retention (Tozuka et al., 2010). In addition, perinatal WD feeding (45% kcals fat) had no effect on MWM spatial memory retention in male and female offspring tested at an older age (20 months), but did appear to rescue the memory deficits in the MWM task induced by unpredictable prenatal stress (a combination of restraint, swimming, wet bedding, noise, food restriction, and lights on overnight) (Bengoetxea et al., 2017). Pavlovian fear conditioning in adult male and female offspring was also not affected by a 60% kcals fat WD during the perinatal period (Peleg-Raibstein et al., 2012). Similarly, maternal consumption of a 43% kcals fat diet had no impact on both contextual and cued fear conditioning in 9.5 months old male offspring (Zieba et al., 2019). While the aforementioned studies suggest that perinatal exposure to diets that are 57–60% kcal fat do not have an effect on spatial memory, one study found that a 60% kcal HFD, given to dams during pregnancy and lactation, resulted in impaired cognitive performance in the NOR task and the Barnes Maze in adult male rats even after being provided a standard diet at PN 21 (Cordner et al., 2019). Overall, these studies provide mixed evidence that perinatal exposure to a WD impairs hippocampal-dependent spatial memory in offspring, highlighting a need for further investigation to identify critical mediating variables.

In addition to the type of learning and memory being evaluated, one major difference between the aforementioned studies that found effects of perinatal WD on various types of memory vs. those that did not is the ratio of fat and sugar content of the WDs. Those that found no effect of perinatal WD exposure on hippocampal-dependent memory utilized diets that were extremely high in fat (around 60% kcal from fat), whereas those that found impaired odor-based avoidance memory utilized a more moderate WD with regard to fat calorie % (45% kcals fat, respectively). In order to have higher fat content in rodent diets, normally this is achieved by reducing the carbohydrate content, which in a WD is typically predominantly sugar. For instance, a 45% kcals fat WD would have higher sugar content (∼17% kcals sucrose) than a 60% kcals fat WD containing ∼7% kcal sucrose. In fact, studies investigating the effects of perinatal WD on spatial learning and memory that used a more moderate WD with regards to fat content (39% kcal from fat) showed that male rats had impaired spatial memory retention in the MWM task, but this was only the case when the animals were maintained on the WD into adulthood as opposed to animals that had their WD replaced with standard chow after lactation (Lépinay et al., 2015). In another study where perinatal exposure to a 45% kcals fat WD was initiated 1 month before mating and continued throughout gestation and lactation, male rat offspring had impaired spatial memory retention in the MWM as adults regardless of whether they were weaned onto standard chow or maintained on the WD (Page et al., 2014). Interestingly, a perinatal diet high in sucrose (20% w/v sucrose solution), but not fat, given to the rat dams from gestational day 1–21 was not sufficient to promote spatial memory impairment in the MWM in either adolescent or adult male offspring (Kuang et al., 2014). Thus, the fat/sugar ratio may have a significant role in determining if learning and memory is impacted, with perinatal exposure to a 45% kcals fat, 17.5% kcals sucrose during the entire perinatal period being particularly detrimental to learning and memory in adulthood.

The ratio of sugar and fat in the diet is also a critical variable for WD effects on metabolism and neuronal outcomes. For instance, odor memory impairment induced by perinatal exposure to a 45% kcals fat WD is associated with dendritic atrophy in the BLA and the CA1 region of the HPC during adulthood (Janthakhin et al., 2017) as well as a reduction in dendritic spines and dendritic length in the mPFC at weaning (Rincel et al., 2018) in male rats. While the ratio of sugar to fat was not specified, mPFC dopamine metabolism was reduced and serotonin metabolism in the mPFC was increased in male and female rats that were exposed to a cafeteria diet perinatally during lactation (Moreton et al., 2019). These results are independent of the obesogenic effects of the diet, as the time on the diet was insufficient to promote obesity in the dams or the offspring who were being tested in a few of aforementioned studies (Janthakhin et al., 2017; Rincel et al., 2018). However, the offspring of rat dams on a 45% kcals fat WD showed reduced hippocampal protein expression of an array of genes that are associated with synaptic plasticity and spatial memory, including BDNF, activity-regulated cytoskeletal-associated protein (Arc), nerve growth factor, synaptophysin, and the NR2B subunit of the glutamate NMDA receptor in adulthood (Page et al., 2014). Cordner et al. found that a 60% kcal perinatal HFD resulted in rats having increased body weights throughout their lifetime as well as having decreased expression of the leptin and insulin receptor in the dentate gyrus and CA3 region of the dorsal hippocampus of offspring at PN 21, which persisted at PN 150, long after the HFD was removed (Cordner et al., 2019). Together, these studies highlight neurobiological mechanisms through which perinatal WD consumption may impact the BLA, mPFC, and HPC to contribute to impaired learning and memory, outcomes that may or may not coincide with poor metabolic outcomes.

In studies that found that learning and memory processes were not impaired following exposure to a perinatal WD, in some cases there were still long-term effects on metabolism and on the brain. For example, despite showing better spatial memory retention, male rat offspring of obese dams on a 60% kcals fat WD exhibited increased neuroinflammation and microglial activation in the HPC (Bilbo and Tsang, 2010). Lack of impairments in fear conditioning in male progeny from dams fed a 43% kcals fat diet was also associated with increased body weight at adulthood (Zieba et al., 2019). In the ventral HPC, male mice subjected to a perinatal 60% kcals fat WD had increased mRNA expression of 5-HT1AR and GABAa alpha2 receptor, suggesting differences in the GABAergic and serotonergic systems despite there not being a group effect on Pavlovian fear conditioning (Peleg-Raibstein et al., 2012). While a perinatal high sucrose diet (20% w/v solution) did not impact spatial memory, exposure to the diet led to increased plasma levels of glucose in the dams, increased body weight in the offspring, and increased apoptosis and activated caspase-3 in the HPC (Kuang et al., 2014). In addition, although maternal consumption of a 60% kcals fat diet did not alter progeny’s cognitive performance in the NOR and Y-maze tasks, these animals still presented greater anxiety-like behaviors and hippocampal inflammation (Winther et al., 2018). Thus, in some cases where perinatal WD exposure is not associated with learning and memory impairments, perinatal exposure to a high fat or high sucrose WD nevertheless leads to metabolic impairments and neurobiological alterations in the HPC, the consequences of which may influence learning and memory at time points later in life that were not investigated in these studies. Regardless, these studies support a framework in which the effects of perinatal WD exposure on learning and memory are largely dissociable from the effects of WD on obesity and associated metabolic dysfunction.

In some cases, the detrimental aspects of the perinatal WD exposure on brain and behavioral outcomes are reversed during adulthood after dietary intervention. For example, the oxidative stress, lipid peroxidation, and decreased BDNF protein levels in the HPC observed following perinatal 57% kcals fat WD exposure were normalized in adulthood when animals were weaned on healthy chow (Tozuka et al., 2010), corresponding to improvements in Barnes Maze memory performance. Together, data highlighted in this section suggest that WD exposure during the perinatal period may negatively impact learning and memory function and HPC neurobiological signaling pathways during adulthood, particularly when the diet contains high percentages of fat and sugar, and even in the absence of obesity. Furthermore, the mixed evidence on learning and memory outcomes after perinatal HFD exposure may depend on the impact maternal HFD exposure has on programming the neural correlates underlying learning and memory and whether or not the maternal programming persists in adulthood. The conditions required for reversal of these long-term disturbances requires further investigation.

### Adolescent WD Consumption

Evidence from multiple studies suggests that adolescent consumption of a WD containing a high % kcal from fat impairs hippocampal-dependent learning and memory in rodents, and that these effects even occur following acute exposure. For example, short-term feeding (1 week, from PN 21–28) of a WD containing 60% kcals from fat in male mice impaired spatial memory in the Y-maze alteration task and object recognition memory impairment in the NOR task during adolescence (Kaczmarczyk et al., 2013). Similarly, impaired object location memory and impaired hippocampal long-term potentiation was reported in adolescent male rats with a similar dietary exposure (Khazen et al., 2019). Finally, impaired extinction of cued fear conditioning is observed in male rats after only 1 week of exposure (PN31–38) to a 41% kcals saturated fat diet in male rats (Vega-Torres et al., 2020). Together, these reports suggest that short-term exposure to high-fat WDs post-weaning impairs spatial and episodic memory during adolescence.

While the aforementioned short-term WD exposure studies suggest that the diet significantly impacted memory, these impairments were likely independent of metabolic effects, as the short duration on the diet was insufficient to promote weight gain (Kaczmarczyk et al., 2013; Khazen et al., 2019; Vega-Torres et al., 2020) or aberrant glucose metabolism (Kaczmarczyk et al., 2013; Vega-Torres et al., 2020). Kaczmarczyk and colleagues reported impaired performance in the NOR task in adolescent mice after both 1 and 3 weeks of exposure to a 60% kcals fat WD, which could be improved by switching animals to a healthy low-fat diet for 1 week. On the other hand, spatial memory deficits in the Y-maze task were present after 1 week, but this effect could no longer be observed after 3 weeks of WD exposure. The 3 weeks of WD timepoint coincides with elevated activity of monoamine oxidase A and B, the enzymes that metabolize dopamine, in the HPC and hypothalamus. In combination with decreased levels of hypothalamic dopamine and increased levels of its metabolic homovanilic acid in the HPC at the 1 week timepoint only, these results suggest spatial memory deficits may be consequent to reduced dopamine signaling after 1 week of WD exposure, whereas dopamine levels are restored after 3 weeks with increased activity of dopamine metabolizing enzymes (Kaczmarczyk et al., 2013). Another possible mechanism for memory impairment following short-term WD feeding involves glucocorticoid receptors. Khazen and colleagues found that intraperitoneal treatment with a glucocorticoid receptor antagonist was able to reverse impaired long. term potentiation and memory deficits, suggesting that glucocorticoid signaling may mediate the effects of WD on hippocampal dysfunction. Similarly, Vega-Torres and colleagues reported dampened neuronal activity in the amygdala following foot shock delivery, as well as increased gene expression for the corticotropin release hormone receptor-1 within the mPFC. In sum, short-term exposure to a WD post-weaning can impair memory independent of the obesogenic effects of the diet, and these memory deficits are associated with changes in dopamine, glucocorticoid signaling, and long-term potentiation in the HPC and mPFC.

Given that short-term feeding of WD impacts memory function, it is not surprising that long-term WD feeding also impairs memory. For example, while binging on a WD (45% kcals from fat) for 2 hrs daily throughout adolescence did not result in spatial memory deficits (Blanco-Gandía et al., 2019), *ad libitum* access (for 1+ month) to this diet after weaning promotes deficits in spatial learning in the MWM task (Boitard et al., 2014), the NOL task (Del Rio et al., 2016), the radial arm maze task (Boitard et al., 2012), and the Hebb Williams Maze (Blanco-Gandía et al., 2019) in adult male rodents. Impairments are also seen in reversal learning in the MWM (Boitard et al., 2014) and enhanced aversive and auditory fear memory (Boitard et al., 2015), as assessed by COA and auditory fear conditioning, respectively. Although 2 h daily access to high fat and high sugar pellets for 28 days during adolescence did not affect odor recognition, rats under this diet regimen failed to demonstrate novelty preference in the NOR task (Reichelt et al., 2020). Adolescent (PN28–56) consumption of a 63% kcal fat diet in male mice impaired discrimination in the Y-maze, reversal learning in the MWM, and cued fear extinction (Labouesse et al., 2017). Alterations in fear extinction were also reported in male rats fed a 41% kcal fat diet for 82 days (PN28–110) (Vega-Torres et al., 2018). Interestingly, adolescent exposure to a lard-enriched WD for 13 weeks (well into adulthood) showed memory deficits in the radial arm maze and the NOL task, despite animals undergoing a 70% caloric restriction over the last 5 weeks of the diet period (Valladolid-Acebes et al., 2011, 2013). Importantly, in some cases switching from a 45% fat diet to a standard rodent diet for 2 weeks can reverse the spatial memory deficits (Blanco-Gandía et al., 2019). Similarly, chronic consumption of a WD initiated during adolescence and consisting of powdered chow, lard, and dextrose (with 41.7% of the calories were derived from fat) is also associated with episodic memory impairments in adulthood (Marwitz et al., 2015). Switching the rats to a control diet for 5 months after an initial 3 months of exposure to a lard-enriched 45% fat WD initiated at weaning, normalized memory impairments in the MWM and COA task (Boitard et al., 2016). These data suggest that male rats develop impairments in spatial memory, reversal learning, and aversive and auditory fear memory in adulthood in response to long-term high-fat, high-sugar consumption starting during adolescence, and moreover, that these effects may be reversible in some cases with dietary intervention. Importantly, learning and memory deficits in male rodents were not observed when WD consumption for a similar duration was confined to adulthood, despite similar diet-induced elevations in body weight and metabolic disruption (Boitard et al., 2012, 2014, 2015; Valladolid-Acebes et al., 2013; Labouesse et al., 2017). These findings corroborate that adolescence is a developmental period of particular vulnerability for WD effects on learning and memory function.

In addition to memory impairment, long-term feeding of a WD also leads to significant disruptions in metabolism and neurobiological systems associated with memory control. While short-term feeding of a WD during adolescence does not promote weight gain, long-term feeding from adolescence to adulthood typically promotes weight gain in male rodents (Valladolid-Acebes et al., 2011, 2013; Boitard et al., 2012, 2014, 2015; Marwitz et al., 2015; Labouesse et al., 2017; Vega-Torres et al., 2018; Blanco-Gandía et al., 2019). Additionally, prolonged intake (3+ months) of a WD started during adolescence and maintained well into adulthood in male rodents imparts metabolic alterations in adulthood such as increased circulating leptin (Valladolid-Acebes et al., 2011, 2013; Boitard et al., 2012), corticosterone, cholesterol, and insulin (Boitard et al., 2012) as well as hyperglycemia (Valladolid-Acebes et al., 2013; Vinuesa et al., 2016) and insulin resistance (Marwitz et al., 2015; Vinuesa et al., 2016). Memory deficits following adolescent WD exposure are also found in the absence of significant weight gain, such as impaired NOR in male rats with intermittent access (2 hrs daily) to high fat and high sugar pellets (Reichelt et al., 2020). Long-term consumption of a WD, initiated during adolescence, resulted in molecular alterations in the HPC, amygdala and mPFC that accompanied memory impairments at adulthood. For example, reduced neurogenesis (Boitard et al., 2012, 2016; Vinuesa et al., 2016), increased microglial activation (Vinuesa et al., 2016) and diminished gene expression of monoamine oxidase A (Reichelt et al., 2020) can be observed in the HPC of rodents fed a WD since adolescence and displaying spatial memory deficits. Alterations in aversive and auditory fear memory in adult male rats fed a WD since adolescence are attenuated by glucocorticoid receptor antagonism in the amygdala (Boitard et al., 2015). Rodents with impaired extinction and reversal learning, but also spatial memory and NOR, due to WD exposure during adolescence, presented with downregulation of the synaptic modulator reelin and altered long term depression (Labouesse et al., 2017) and reduced BDNF and monoamine oxidase A gene expression (Reichelt et al., 2020) in the mPFC. Altogether, these results demonstrate weight gain and metabolic impairments often accompany but are not conditional for WD initiated during adolescence to induce learning and memory deficits. These data further suggest neurogenesis, microglial activation, glucocorticoid signaling, as well as synaptic transmission and neural plasticity in the HPC, amygdala and mPFC as potential mechanisms.

Given that rodent WDs high in fat are often also high in sugar, it is important to investigate the contribution of dietary sugars to the effects on learning and memory. Common obesity-promoting diet compositions from Research Diets consist of the 45% kcal HFD, which contains 17.5% of kcal from sucrose, the 58% kcal HFD, which contains about 13% of kcal from sucrose, and the 60% kcal HFD, which contains about 7.5% of kcal from sucrose. Interestingly, the effects of sugar alone have been shown to impact learning and memory independent of weight gain when given during the adolescent period (Hsu et al., 2015; Reichelt et al., 2015; Abbott et al., 2016; Alten et al., 2018; Buyukata et al., 2018; Noble et al., 2019). Furthermore, the effects of adolescent dietary sugar on learning and memory function persist into adulthood. For example, male rats given free access to an 11% w/v high fructose corn syrup drink for at least 30 days during adolescence had episodic and spatial memory impairments, assessed by the NOIC, Barnes Maze, and Morris Water Maze tasks (Hsu et al., 2015; Alten et al., 2018; Noble et al., 2019). Furthermore, NOIC memory impairments persisted even when animals were tested after several months without access to sugar solutions (Noble et al., 2019). Notably, adult rats fed sugar solutions for a similar length of time did not show memory deficits (Hsu et al., 2015). Rats consuming a high in sugar, but low in fat diet (26.7% sucrose/lactose, 6.5% fat) starting at weaning impaired episodic and spatial memory in the object recognition and Y-Maze tasks and impaired learning in the contextual fear conditioning task in adulthood (Altermann Torre et al., 2020). Overall, these studies suggest that WDs high in sugar (independent of elevated fat content vs. a control diet) have adverse effects on learning and memory that last into adulthood and are not easily reversible by removal of the diet.

While the studies described above typically involve *ad libitum* access to the experimental diet, some studies have examined the effects of intermittent access to a sugar solution during adolescence on learning and memory function. Results of these studies suggest that intermittent sugar access similarly conferred lasting impairments in learning and memory function later in life (Reichelt et al., 2015; Abbott et al., 2016; Buyukata et al., 2018; Noble et al., 2019). For example, male rats given intermittent access (2 hrs daily) to a 10% w/v sucrose drink during adolescence were impaired in both the place recognition (Abbott et al., 2016) and object-in-place recognition tasks (Reichelt et al., 2015; Abbott et al., 2016) and were unable to use contextual information to discriminate between the context-appropriate and context-inappropriate levers in a context devaluation task, which requires communication between the mPFC and HPC (Reichelt et al., 2015). Similarly, male rats with intermittent access to a 10% sucrose solution for 28 days (PN28–55) presented impairments in both learning and memory in the MWM task at adulthood (Kendig et al., 2013). Despite conferring impairments in learning and memory, male rats that had free access to an 11% w/v sugar solution have normal body weights throughout the dietary exposure period (Hsu et al., 2015; Noble et al., 2019), with one study finding that consumption of a high fructose corn syrup solution actually led to a decrease in body weight despite the rats showing glucose intolerance and increased adiposity (Alten et al., 2018). Intermittent access to an 10–11% w/v sugar solution also did not promote weight gain during the 30 days of access in either males (Kendig et al., 2013; Abbott et al., 2016; Noble et al., 2019) or females (Abbott et al., 2016), yet one study has found that significant weight gain occurred in male rats after the intermittent access period to a 10% sucrose solution (Reichelt et al., 2015). Collectively, these studies show that intermittent access to a sugar solution during adolescence can impart long-lasting memory deficits, and that these effects can occur independent of body weight gain.

The influence of sex with regards to adolescent WD exposure effects on memory function is poorly understood. Males, but not females, on a chronic 45% kcals fat WD have reduced freezing behavior compared with chow fed controls in a contextual fear conditioning task in adulthood (Hwang et al., 2010). However, given that WD consumption can be anxiolytic, it is difficult to determine whether the reduced freezing behavior was due to improved memory function *per se*, or was a function of reduced anxiety. Moreover, Buyukata et al. found that both male and female rats on an intermittent sucrose access schedule showed impaired NOR memory during adolescence when the objects shared multiple similar features. However, when the objects were arranged with either small or large spatial separations (spontaneous location recognition task), males that consumed sugar performed worse in tasks with small spatial separations, whereas females performed worse in tasks with large spatial separations (Buyukata et al., 2018). In another study, both male and female rats previously on a chronic 58% kcals fat WD during adolescence displayed impaired memory in a spatial object recognition task in adulthood (Underwood and Thompson, 2016). In addition, female mice fed for 12 weeks of a 60% kcals fat WD showed altered reversal but not initial learning in the MWM task (Klein et al., 2016). While these studies suggest that the effects of adolescent WD exposure are sexually dimorphic depending on the task and type of memory being tested, further research is clearly needed in this area.

Estrogen may be a critical factor mediating sex differences in vulnerability to adolescent WD-induced memory impairments. For example, in female rats intermittent dietary sucrose access during adolescence (10% w/v sucrose solution, 2 hrs daily) did not impact NPR performance, however the rats were only able to perform place recognition correctly during the proestrus phase of the estrous cycle, a stage that contains higher levels of circulating estrogens (Abbott et al., 2016). Taken together, similar to males, female rats given intermittent sugar access display impairments in episodic memory. However, the episodic memory deficits may be determined by the stage of the estrous cycle.

Metabolically, female mice exhibit similar deficits to males in response to a 45% kcal from fat WD from adolescence to adulthood, having significant weight gain relative to controls despite comparable caloric intake (Hwang et al., 2010). However, WD-fed males are distinguished from females by having higher glucose levels relative to controls fed a healthy low fat diet (Hwang et al., 2010). Unlike male rats, female rats did not gain significant weight or display glucose intolerance after chronic WD exposure (58% kcal fat), suggesting that female rats may develop a less severe metabolic phenotype under these conditions compared to males (Underwood and Thompson, 2016). However, female mice fed a 60% kcal fat WD for 12 weeks did develop hyperphagia and greater body weight gain relative to animals receiving the control diet, and alterations in reversal learning in the MWM task were prevented by wheel running (Klein et al., 2016). As for a potential mechanism as to how male and female rodents differ in regard to contextual fear conditioning, Hwang et al. (Hwang et al., 2010) found that WD-fed males, but not females, had reduced long term potentiation but were also lacking a normal long term depression response. Together, these studies suggest that chronic exposure to WD starting in adolescence may alter learning and memory processes in female rodents, although the specific types of memory involved may be sex-dependent, and like males, memory function is dissociable from metabolic impairments.

With regard to underlying neurobiological mechanisms for how high sugar diets during adolescence impact memory function, memory deficits induced by free access to the 11% w/v sugar solution in adolescence are associated with increased plasma insulin and pro-inflammatory cytokines such as interleukin 6 and interleukin 1β in the dorsal HPC (Hsu et al., 2015). Moreover, another study found systemic inflammation after adolescent consumption of a 11% w/v sugar solution. Using in-vivo electrophysiology, the authors revealed that concurrent with systemic inflammation, high fructose corn syrup consumption induced hyperexcitability in hippocampal CA3-CA1 synapses (Alten et al., 2018). Furthermore, the effect of a diet high in sugar on plasticity depended on the developmental stage, such that during adolescence, 1 week of consumption reduced synaptophysin, BDNF, protein kinase B (AKT), and phosphorylated AKT in the HPC. However, when access to the simple sugar-enriched WD is maintained into adulthood, there is increased synaptophysin, spinophilin/neurabin-II, and decreased BDNF and neuronal nitric oxide synthase, suggesting that plasticity markers change depending on stage of development (Torre et al., 2020). As opposed to free access to sugar, the memory deficits associated with intermittent access to sugar in adolescent males were accompanied by deficits in parvalbumin-immunoreactive cell density in the HPC and mPFC in adulthood (Reichelt et al., 2015). Altogether, these findings suggest that the HPC is a region that is particularly sensitive to perturbations by adolescent dietary sugar consumption, with plasticity and inflammatory signaling pathways implicated as putative mechanistic links between diet and memory dysfunction.

Cafeteria diets are often obesogenic when consumed by rodents during adulthood, however, whether or not adolescent consumption of cafeteria diets in rodents promotes cognitive impairment is still controversial. For example, adolescent male rats on a cafeteria diet consisting of a variety of high fat and high sugar palatable food options, standard chow, and a 15% (w/v) sucrose solution weighed more and had greater adiposity compared with rats maintained on chow and water alone. The cafeteria diet-fed rats also exhibited impairments in hippocampal-dependent spatial learning and memory in the MWM, but not object novelty detection or fear acquisition, during adulthood (Ferreira et al., 2018). In contrast, a similar study, using very similar dietary parameters of highly palatable human foods along with standard chow and a 12% w/v sucrose solution, found no effect of adolescent consumption of a cafeteria diet on spatial memory in adulthood using the Barnes Maze (Gomez-Smith et al., 2016). Despite intact spatial learning, the cafeteria diet rats displayed an obesogenic phenotype as indicated by increased body weights, visceral adiposity, hyperinsulinemia, glucose intolerance, and dyslipidemia with elevated serum triglyceride levels and reduced HDL cholesterol, and greater hippocampal neuroinflammation in adulthood. Moreover, replacing the cafeteria diet with a standard rodent diet appeared to reverse all of the metabolic deficits mentioned before as well as the neuroinflammation (Gomez-Smith et al., 2016). These studies reveal that the effects of an adolescent cafeteria diet on memory are variable and highlight that more work is needed to identify critical mediating variables. However, given that in some cases obesogenic effects have been observed in the absence of memory impairments (and vice versa), these findings further highlight a framework in which the effects of early life WD consumption on cognition and metabolism are dissociable.

### Summary

**FIGURE 2:**
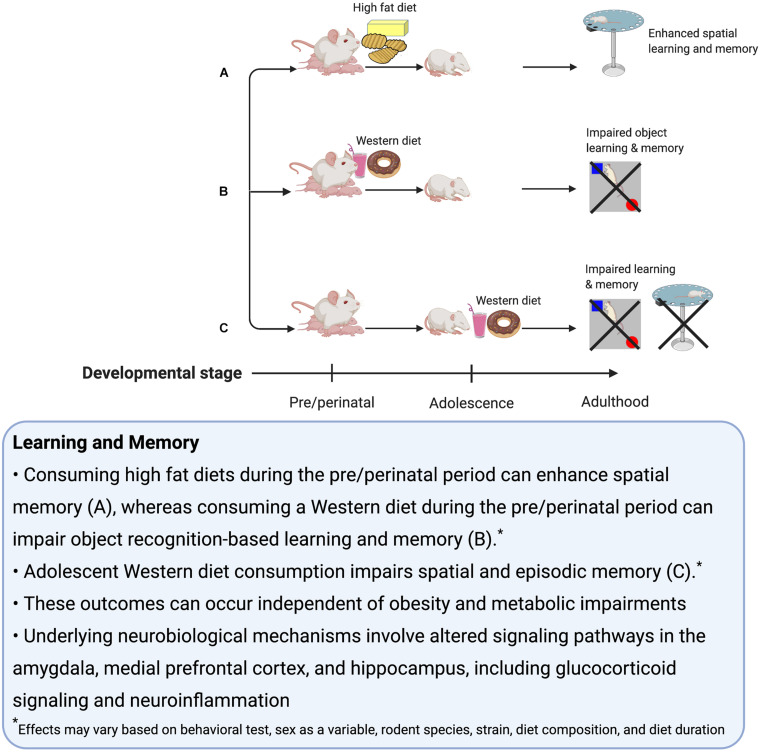
Summary of the effects of early life Western diet consumption on learning and memory.

Adolescent exposure to WD factors contributes to memory impairments, even in the absence of weight gain (Figure 2). While our focus here is on rodent models, studies have identified a deleterious impact of early life WD consumption on memory function in humans as well (Baym et al., 2014; Haapala et al., 2015; Khan et al., 2015; Cohen et al., 2018). Given that the rodent and human HPC have similar development patterns during the adolescent period, the insights garnered from rodent studies may provide insight into how WD factors might impact human adolescent brain development. The studies discussed in this section further highlight that rodent models have elucidated potential mechanisms for the effects of WD on learning and memory impairments, and these relate to region-specific changes in Ca2+ dysregulation (long term potentiation), glucocorticoid receptors, dopamine metabolism, neuroinflammation, microglial activity, and other factors that affect plasticity and ultimately alter the network dynamics of neural ensembles that support cognition, with the HPC, mPFC, and amygdala being particularly affected. In conclusion, several studies have identified adolescence as a period of high vulnerability for deleterious effects of WD consumption on memory and neural processes associated with memory control. More research is needed to fully understand the extent that sex and sex hormones are critical variables, as well as the effectiveness of various interventions (dietary, etc.) to reverse the long-lasting memory impairment associated with early life WD consumption.

## Reward-Motivated Behavior

### Behavioral Models and Neural Substrates

Consumption of palatable food such as a rodent WD engages hedonic- and reward-associated neural systems, and therefore it is not surprising that early-life WD consumption can have lasting impacts on these systems and associated behaviors (Corsica and Hood, 2011; Tompkins et al., 2017; Lowe et al., 2020). Alterations in reward-associated neural systems affect susceptibility to binge eating (Ames et al., 2014; Bodell et al., 2018), obesity (Matton et al., 2013), and addiction to substances of abuse (De Cock et al., 2016). In rodent studies, many behavioral tasks are commonly utilized to investigate these neural reward systems. Those discussed in this section include: sensitization, defined as an increase in locomotion following repeated administrations of drugs that upregulate dopamine signaling; conditioned place preference (CPP), which measures the strength of an association between a rewarding stimulus (e.g., palatable food consumption) and a context; the operant runway task, which measures the time it takes for the animal to reach the goal area and obtain a food reward (Kuhn et al., 2019); visual discrimination reversal learning and attentional set shifting, which are tests of behavioral flexibility (Heisler et al., 2015); operant responding on a progressive ratio reinforcement schedule, which tests for motivation to work for a reinforcer (Killeen et al., 2009); Pavlovian Conditioned Approach where animals show appetitive responses to cues that predict a food reward (Fitzpatrick and Morrow, 2016); outcome devaluation, which tests, among other things, whether or not the animal is exhibiting habitual behavior (Rossi and Yin, 2012); taste reactivity, which indicates an animal’s hedonic evaluation of the taste of a food based on their orofacial reactions; macronutrient preference testing (e.g., carbohydrates vs. fat) (Grill and Norgren, 1978) and monitoring intake during chronic access to palatable food. Some additional behavioral tasks discussed in this section measure impulsive behavior, including: the delay-discounting task, which assesses preference for smaller more immediate over larger more delayed food reinforcers; the 5-Choice Serial Reaction Time Test, which discerns impulsivity (via incorrect trials and premature trials) from inattention (via omitted trials) (Dent and Isles, 2014).

Dopaminergic projections from the ventral tegmental area (VTA) to the ACB are involved in motivated behavior, especially Pavlovian cue-reward associative learning (Saunders et al., 2018). On the other hand, opioid signaling in the ACB, within the ventral striatum, is identified to play a role in linking sweet tastants to hedonic orofacial ‘liking’ reactions (Smith and Berridge, 2007; Berridge, 2009). There is also evidence to suggest in addition to the striatum, the amygdala is critical for reinforcement learning (Averbeck and Costa, 2017). In a broader context, regions activated by palatable foods are nodes within a larger neural network that encompass connections to other regions such as the hypothalamus, mPFC and HPC that modulate behavior related to reward learning, food intake, impulse control, and novelty (Kelley et al., 2005). In this section, we discuss potential mechanisms for how early life obesogenic diets influence reward-motivated behavior, in part through changes in the dopamine and/or opioid systems.

### Perinatal WD Exposure

Perinatal exposure to a WD can dysregulate reward-motivated behavior, even when offspring are reared on a standard chow diet (Naef et al., 2008, 2011; Ong and Muhlhausler, 2011; Morganstern et al., 2013; Wu et al., 2013; Grissom et al., 2015; Peleg-Raibstein et al., 2016; Roversi et al., 2016; Paradis et al., 2017; Sarker et al., 2019; Gawliński et al., 2020). For example, when dams were exposed to a WD (60% kcals from fat) pre-conception there were no differences in sensitivity to amphetamine, as indicated by amphetamine-induced locomotor activity, or preference for alcohol in offspring. However, maternal exposure to a WD diet during late gestation (gestational days (GD) 12–21) increased alcohol preference and amphetamine sensitivity in male and female mice offspring when tested in adulthood (Sarker et al., 2019). These effects likely involve epigenetic mechanisms as behavioral effects carried on into the 3rd generation where females, but not males, still showed elevated addictive-like behaviors if the WD was provided from preconception to weaning (Sarker et al., 2018). However, another study using a WD (30% kcals fat) found the opposite, where a longer exposure from GD 13 to PN 21 resulted in the offspring being less sensitized, as seen by reduced locomotor activity, to both an initial low-dose exposure to amphetamine and with repeated exposure to amphetamine in adulthood (Naef et al., 2008). Subsequent work with similar dietary conditions demonstrated that male offspring from WD-fed dams display blunted ACB dopamine release following amphetamine administration (Naef et al., 2011). This was accompanied by altered D2 receptor function and signaling, as well as increased motivation for fat rewards. Together these studies show that perinatal WD exposure during late gestation can lead to lasting alterations in reward-motivated behavior in the offspring in adulthood, and that the % kcals fat in the WD may be a critical factor.

WD consumption by dams through an extended perinatal period that includes several weeks of pre-conception, gestation, and lactation yields long-lasting effects on reward-motivated behavior. For example, male and female rats born to dams that received hydrogenated vegetable fat (3 g/kg), a trans-fat, by oral gavage during the extended perinatal period preferred a morphine-associated context in CPP during late adolescence (PN 41+) to a larger degree than control female offspring (Roversi et al., 2016). Similarly, male and female offspring from dams consuming a 60% kcals fat WD initiated prior to conception demonstrated enhanced CPP acquisition for cocaine, as well as greater cocaine and amphetamine sensitization (Peleg-Raibstein et al., 2016). In addition, these animals also display a stronger preference for fat, sugar, and ethanol relative to control offspring. Also, when dams are exposed to a WD where 60% of calories are derived from fat throughout the extended perinatal period, male rat offspring were impaired in behavioral flexibility for sucrose reinforcement in the visual discrimination reversal learning and attentional set shifting tasks (Wu et al., 2013). Additionally, a 60% kcals fat WD during gestation and lactation resulted in both male and female mouse offspring displaying decreased motivation to earn a liquid food reward in operant responding under a progressive ratio schedule of reinforcement, as well as increased impulsivity in the 5-Choice Serial Reaction Time task as measured by incorrect and impulsive premature responses. These effects were observed despite the offspring being food restricted to 80–95% of their free-feeding body weights (Grissom et al., 2015). Finally, if a cafeteria diet was provided during the extended perinatal period, male and female rat offspring responded to a junk food challenge later in adolescence and adulthood by increasing their caloric consumption relative to controls (Ong and Muhlhausler, 2011, 2014), suggesting that WD exposure during the extended perinatal exposure leads to increased consumption of palatable food by the offspring later in life.

Even when WD exposure is initiated at the beginning of gestation, reward functions can be disturbed in the offspring. For example, when dams are fed a WD from gestational day 1 until end of lactation, male pups show a transient enhanced fat preference at PN25 that normalizes by PN95 (Paradis et al., 2017). Female offspring of dams fed a high sugar diet (44% kcals from sucrose) were more responsive to cue and cocaine-induced reinstatement of lever pressing (Gawliński et al., 2020). Finally, maternal consumption of a high fat/high sucrose diet from gestation day 5 until end of lactation increased operant responding for nicotine infusion in the male offspring (Morganstern et al., 2013). Altogether, these studies demonstrate that perinatal WD exposure starting as early as before pregnancy and terminating around the time of weaning increases drive for rewards (increased preference for a place associated with reward, impaired behavioral flexibility, increased impulsivity, and increased consumption of palatable if made freely available), with the exception of one study that observed reduced motivation to work for liquid food reward and no differences in Pavlovian conditioned approach behavior (Grissom et al., 2015).

Similar to the effects described in the earlier sections on anxiety, learning, and memory, an obesogenic phenotype is not required for there to be differences in reward-motivated behavior associated with perinatal WD consumption. While some studies observed that a perinatal WD increases body weight and adiposity and promotes disturbances in metabolism (e.g., insulin insensitivity) in offspring at weaning (Naef et al., 2008; Ong and Muhlhausler, 2011; Morganstern et al., 2013; Peleg-Raibstein et al., 2016; Paradis et al., 2017; Song et al., 2020) and as adults (Naef et al., 2011; Ong and Muhlhausler, 2011; Morganstern et al., 2013; Peleg-Raibstein et al., 2016; Paradis et al., 2017; Sarker et al., 2019; Song et al., 2020), other studies found no such differences (Wu et al., 2013; Ong and Muhlhausler, 2014; Grissom et al., 2015; Roversi et al., 2016; Gawliński et al., 2020), even when the dams were heavier as a result of the obesogenic diet (Wu et al., 2013; Ong and Muhlhausler, 2014). Again, these differences could be reflective of the type of WD being used, with those higher in fat leading to more severe metabolic consequences, and/or also the different rodent strains being used. Regardless, all of the aforementioned studies found differences in reward-motivated behavior, with the exception of 2 studies that found no effects in reward-motivated behavior when dams were fed a 60% kcals fat WD (Sarker et al., 2019; Song et al., 2020). Despite fat preference being only affected in offspring from WD dams who had access to a running wheel, juveniles from the WD-sedentary group still displayed changes in dopamine and opioid-related gene expression in the mPFC, ACB and VTA, with some of these changes lasting throughout adulthood (Song et al., 2020). Sarker and colleagues (Sarker et al., 2019) observed no impact of perinatal WD on high fat diet, sucrose and alcohol preference as well as locomotor response to amphetamine in offspring when maternal WD access was confined to lactation. While lactation may be too short of an exposure period to induce behavioral deficits, WD exposure still resulted in weight gain, a higher fat mass ratio, a higher lipid profile, and high insulin insensitivity relative to controls (Sarker et al., 2019), thus further highlighting that the effects of perinatal WD consumption diets on reward-motivated behavior and obesity can be dissociated.

Effects on reward-mediated behavioral outcomes associated with perinatal WD exposure may be driven, in part, by altered striatal dopamine signaling. Studies have shown that the ventral striatum, which contains the ACB, is important for encoding food-related reward value and that dopamine is released in response to food-associated cues (Wyvell and Berridge, 2001; Ostlund et al., 2014; Alonso-Alonso et al., 2015; Kosheleff et al., 2018) whereas the dorsal striatum is related to food craving and consumption (Small et al., 2003; DiFeliceantonio et al., 2012; Contreras-Rodríguez et al., 2017). In the striatal regions of rodents, increased sensitization to amphetamine after gestational WD (gestational day 12 to 21) exposure was accompanied by a hypodopaminergic state where both male and female offspring had reduced dopamine levels in both the ventral and dorsal striatum relative to controls. These outcomes were accompanied by higher levels of dopamine and its metabolites in the VTA, which was suggested to be compensating for the reduced dopamine levels in the striatum (Sarker et al., 2019). In contrast, when reduced sensitization to amphetamine was observed after WD exposure from gestational day 13 to PN 21, male offspring had increased levels of dopamine in both the ventral striatum and VTA (Naef et al., 2008). Together, these findings suggest that WD exposure restricted to the gestational period reduces levels of dopamine in the ventral striatum and increases sensitization, whereas longer-term exposure that includes the gestational period increases levels of dopamine in the ventral striatum and decreases sensitization to amphetamine.

When perinatal WD feeding is extended from 3 weeks prior to conception until end of lactation, hyper-responsiveness to cocaine and amphetamine occurs in a context of reduced basal striatal and VTA dopamine levels, but enhanced ACB deltaFosB expression following cocaine exposure relative to control offspring (Peleg-Raibstein et al., 2016). In addition to highlighting a dissociation between basal content and reactivity, these neurochemical observations were associated with lower content of dopamine metabolites, suggesting molecular adaptations to maintain dopamine signaling. In addition to altered locomotor response to amphetamine, impairments in the visual discrimination reversal learning task after an extended perinatal WD exposure may also be due to reduced dopamine uptake in the dorsal striatum in male offspring, suggesting abnormal dopamine clearance being involved in reversal learning impairment (Wu et al., 2013). Another study found that expression of D1R was lower in the ACB, but higher in the VTA after an extended perinatal cafeteria diet, which may be related to male and female rat susceptibility for increased caloric consumption of junk food in adulthood (Ong and Muhlhausler, 2014). Transient increased fat preference coincides with greater D2R gene expression in the ACB, while normalization of fat preference occurs when VTA and ACB tyrosine hydroxylase expression is downregulated (Paradis et al., 2017). Despite offspring showing enhanced preference for junk food at both time points, ACB gene expression for mu opioid receptor is increased and dopamine transporter is decreased during adolescence, while changes occur in the opposite direction at adulthood (Ong and Muhlhausler, 2011). In addition to facilitating cocaine-induced reinstatement, maternal exposure to a high sucrose diet is associated with higher striatal levels of melanocortin 4 receptor in the adult offspring (Gawliński et al., 2020). Grissom and colleagues found that attention and impulsivity deficits observed after perinatal WD exposure during gestation and lactation were linked with overexpression of DNMT1 in the mPFC, which is involved in DNA methylation, catechol-o-methyltransferase, which is an enzyme that degrades catecholamines like dopamine, δ-opioid receptor, whose activation can lead to increased extracellular dopamine (Burns et al., 2019), and cannabinoid receptor 1, which regulates the expression of D2R (Blume et al., 2013; Grissom et al., 2015). In rat offspring, heightened operant responding for nicotine infusions induced by maternal WD was accompanied by dampened acetylcholinesterase activity in the striatum, VTA and hypothalamus, as well as increased nicotinic acetylcholine receptor binding in the VTA (Morganstern et al., 2013). Finally, oxidative stress in the VTA was seen in male and female rats that showed stronger preference for the morphine-associated side in CPP when they were born to dams that received extended perinatal exposure to trans-fat, even in the animals that received trans-fat exposure without any morphine exposure (Roversi et al., 2016). While not evaluated in the aforementioned study, oxidative stress can lead to the degeneration of dopaminergic neurons and dopamine metabolism (Dias et al., 2013), warranting further research on the association between perinatal WD, oxidative stress, and altered dopaminergic signaling. Altogether, there are several mechanisms that could be involved in the development of reward-motivated deficits associated with perinatal WD exposure, with dopaminergic signaling being a prime suspect.

### Adolescent WD Exposure

Similar to perinatal exposure, adolescent consumption of WDs also impacts reward-motivated behavioral outcomes. For example, adolescent access to a WD, where 40–45% of total kcals are derived from fat, alters diet preference for fat and carbohydrates (Teegarden et al., 2009; Steele et al., 2019) and increases impulsivity (Steele et al., 2019). Specifically, after only 1 week of exposure to a WD (44.9% kcals from fat) at 3 weeks old, WD mice consumed significantly more calories from relative to controls when given a macronutrient choice test, which included options for a high fat diet, a high carbohydrate diet, or high protein diet in adulthood for 10 days. However, this preference did not lead to overconsumption of a WD (44.9% kcals fat) when provided with chronic access (15 weeks) in adulthood (Teegarden et al., 2009). Food preference shifts can also occur without increased hedonic orofacial liking responses in the taste reactivity test, as adolescent male rats exposed to a chronic WD (40% of calories from hydrogenated vegetable fat) for 8 weeks after weaning did not display differences in taste reactivity for fat or sugar, yet still preferred fat over sugar in a preference test in adulthood compared to age-matched rats that received a high sugar diet (40% of the calories from a powdered sugar and water mixture) or a healthy low fat diet, both of which preferred sugar over fat (Steele et al., 2019). Importantly, these animals were habituated to both the sugar and the fat a day before preference testing, so the results were not confounded by the novelty of the foods. In addition to alterations in food preference, both the high fat and high sugar groups were also more impulsive in an impulsive-choice test, where the reward was grain pellets, as indicated by increased delay sensitivity/delay discounting. In addition to having an increased discounting rate for delayed rewards, the high fat diet-fed rats were also more impulsive relative to the high-sugar group and the control group when the magnitude of the reward was manipulated (Steele et al., 2019). On the other hand, 8 weeks of daily access to a cafeteria diet did not impact delay discounting for grain-based pellets, however these animals were more sensitive to the preference for smaller, immediate rewards induced by D2R antagonism (Robertson and Rasmussen, 2017). Another study found that male and female mice exposed to an even higher fat diet (60% kcals fat) for 12 weeks beginning at weaning preferred water over a 4% w/v sucrose solution in adulthood, suggesting possible anhedonia and a hypo-reward response. However this effect dissipated after switching to a healthy low fat diet for 4 weeks (Carlin et al., 2016). Accordingly, exposure to a WD reduced sucrose and saccharin preference during adolescence, a phenotype that was no longer observed at adulthood (Rabasa et al., 2016). These studies indicate that adolescent consumption of a WD increases impulsivity for food and dietary preference in adulthood, with adolescent exposure to a high fat leading to increased preference for a high fat diet in adulthood. Additional studies where chronic access is reintroduced in adulthood to determine whether or not these preferences influence long-term dietary choices would be intriguing.

In addition to increasing impulsivity and altering food choice, consuming a WD during adolescence affects motivation for food rewards (Tantot et al., 2017) and increases drug-seeking behavior (Blanco-Gandía et al., 2017; Naneix et al., 2017). For example, long-term exposure to a WD (45% kcal fat) from weaning and continuing into adulthood in male rats alters appetitive instrumental behavior such that WD-exposed rats show reduced motivation for food rewards (grain or sucrose) when under mild deprivation (90% of their *ad libitum* feeding body weight) in a progressive ratio test, where reinforcement during training was based on a random interval, but not random ratio, schedule (Tantot et al., 2017). Tantot and colleagues also found that training on a random interval schedule led WD-exposed rats to develop habitual food-seeking behavior faster than control rats, based on results from an outcome devaluation task (Tantot et al., 2017). However, both of these effects are reversed by subsequent training on a random ratio schedule. Furthermore, consuming a WD higher in fat (60% kcals fat) during adolescence impaired CPP for a food reward high in fat, despite consuming more of the reward during training, which was taken into consideration since the rats did not finish consuming the food in any given trial (Privitera et al., 2011). Importantly, the authors found that adult rats subjected to a 60% kcals WD in adulthood displayed normal CPP for the high fat reward, highlighting that CPP was only impaired when the high fat WD was consumed during adolescence. In the case of drug rewards, however, others have found that mice maintained on a WD (45% kcals fat) where access was given for 2 h/day thrice weekly since adolescence (PN 29) increased sensitivity to the reinforcing effects of a subthreshold dose of cocaine in the CPP task in adulthood (Blanco-Gandía et al., 2017). However, rats maintained on an *ad libitum* 40% kcals fat WD since adolescence (PN 21) had no impact on CPP acquisition for cocaine in adulthood, although this result may have been confounded by the rats being placed on significant water restriction (20 min access per day) during behavioral training and testing (Clasen et al., 2020). Further, when given the opportunity for intravenous self-administration of cocaine, WD-fed male mice elevated levels of self-administration. Ad libitum WD (45% kcals fat) exposure from weaning to adulthood can also lead to heightened amphetamine sensitization at adulthood in male rats relative to rats fed a control diet (Naneix et al., 2017). Taken together, these results suggest that chronic experience with consuming a WD with at least 45% of energy provided by fat starting at adolescence can lead to impaired goal-directed actions for food rewards and increased sensitivity to psychostimulant drugs in adulthood.

As WDs high in fat often contain a higher kcal percentage of sugar compared to control diets, it is important to consider the specific role of sugar in WD-associated effects on reward-motivated behavior. Indeed, there are studies suggesting that sugar affects reward-motivated behavior independent of elevated fat content in the diet (Frazier et al., 2008; Vendruscolo et al., 2010; Kendig et al., 2013; Naneix et al., 2016, 2018; Gueye et al., 2018; Steele et al., 2019). For instance, male rats on a 5% w/v sucrose solution during adolescence (PN 30–46) showed reduced motivation to obtain the low-calorie sweetener saccharin (sweet reinforcer) and maltodextrin (non-sweet carbohydrate reinforcer), but not cocaine in adulthood, as assessed by performance on fixed- and progressive-ratio schedules of reinforcement (Vendruscolo et al., 2010). These findings are supported by another study reporting diminished operant responding for saccharin under a progressive ratio schedule of reinforcement, as well as blunted sucrose preference in adults who had access to a 5% sucrose solution for 16 days during adolescence (Gueye et al., 2018). Adolescent exposure to 5% sucrose solution in male rats also resulted, at adulthood, in reduced intake of either saccharin or sucrose compared to water during two-bottle choice tests, as well as a decrease in positive ‘liking’ orofacial reactions to sucrose or saccharin relative to controls (Naneix et al., 2016). While there was no effect of brief adolescent access to a 5% sucrose solution, adults rats previously exposed to the sucrose during adolescence showed blunted sensitivity to D1R and D2R pharmacological manipulations on operant responding for 0.13% w/v saccharin solution at adulthood (Naneix et al., 2018). Furthermore, chronic access to *ad libitum* sucrose pellets in male and female mice starting at weaning for 4–7 weeks before being maintained on standard chow reduced motivation for sucrose under a progressive ratio schedule, but there were no differences in sucrose preference over water, nor differences in the amount of food consumed with a 3-week high-sugar, high-fat dietary challenge relative to controls (Frazier et al., 2008). This contrasts with findings in male rats, where a chronic high-sugar diet (∼40% kcals consumed from liquid sugar solution, ∼60% kcals consumed from standard chow for 8 weeks starting at PN 21), promoted a choice consumption bias for sugar over fat without differences in taste reactivity for sugar or fat (Steele et al., 2019). On the other hand, intermittent access to a 10% w/v sucrose or 0.1% w/v saccharin solution during adolescence failed to alter operant responding and delay discounting for food rewards, however, unlike the previous studies, reward pellets were sugar-enriched grain-based rather than pure sucrose (Kendig et al., 2013). The aforementioned studies show that adolescent WDs high in sugar reduce motivation for sweet taste in adulthood and lead to differences in preference for sweet taste, with some rodents also displaying decreased positive orofacial reactions to sweet taste. However, this may be dependent on the food choices available given that when sugar was provided during adolescence as a sweet taste preference was altered relative to control animals fed a healthy diet when sugar was offered as a beverage but not as a solid food.

While there are clear impacts of WD consumption during adolescence on reward-motivated behavior in rodents, obesity (or even increased body weight) is not required for these effects to occur. For instance, rodents displaying altered reward-motivated behavior following adolescent access to sugar either with a 5% w/v sucrose solution, sucrose pellets, or with a 40% kcals sugar diet had similar body weights relative to controls (Frazier et al., 2008; Vendruscolo et al., 2010; Naneix et al., 2016, 2018; Gueye et al., 2018; Steele et al., 2019). These outcomes are mirrored by comparable total energy intake, as the rats in these studies compensate for sugar calories by reducing chow intake (Vendruscolo et al., 2010; Naneix et al., 2016, 2018). However, after a high fat, high sugar WD challenge in adulthood, rats previously exposed to *ad libitum* sucrose pellets during adolescence gained more weight relative to controls (Frazier et al., 2008). Animals on a WD during adolescence or from adolescence to adulthood who showed reward impairments, either gained substantial weight relative to controls ((Privitera et al., 2011; Carlin et al., 2016; Rabasa et al., 2016; Naneix et al., 2017; Robertson and Rasmussen, 2017; Tantot et al., 2017) or did not (Teegarden et al., 2009; Blanco-Gandía et al., 2017), with less exposure (i.e., thrice weekly binge model or a 1 week exposure) likely contributing to normal weight gain in the latter studies. In addition to weight gain and reward deficits, rats fed a WD (45% kcals fat) through adolescence and early adulthood showed increased levels of leptin, insulin, and cholesterol, but not triglycerides (Naneix et al., 2017; Tantot et al., 2017) with one study finding increased adiposity (Carlin et al., 2016). Even though intermittent WD access (2 hrs daily, thrice weekly) during adolescence and early adulthood did not increase body weight, alterations in cocaine CPP in these male mice was still accompanied by greater plasma ghrelin levels (Blanco-Gandía et al., 2017). Altogether, these studies show that an obesogenic phenotype is not required for adolescent onset high sugar or high fat WD exposure to have an effect on reward-mediated behavior in rodents, yet alterations in reward processes may drive WD consumption and weight gain and metabolic alterations later in life.

Similar to perinatal WD exposure, adolescent WD consumption influences reward-motivated behaviors, in part, via alterations mesolimbic dopamine, cannabinoid and opioid signaling. For example, WD-exposed rodents that showed increased locomotor sensitization to amphetamine displayed a concomitant sensitized mesolimbic dopamine response as reflected by increased bursting activity of dopaminergic neurons in the VTA and increased ACB c-FOS expression (indicative of increased neural activation) in response to amphetamine administration. The WD-fed rats also showed increased dopamine release and tyrosine hydroxylase and D2R expression in the ACB (Naneix et al., 2017). On the other hand, similar exposure to sucrose during adolescence reduced D1R and D2R protein expression in the ACB under basal conditions (Naneix et al., 2018). Similarly, male mice that binged on a 45% kcals WD for 2 h a day from adolescence to adulthood were more sensitive to the reinforcing effects of another dopamine-enhancing drug, cocaine, as assessed by CPP and drug self-administration (Blanco-Gandía et al., 2017). These mice had reduced cannabinoid receptor 1 and mu opioid receptor in the ACB, but greater ghrelin receptor expression in the VTA. Mice that received 1 week exposure to a WD (approximately 45% kcal fat) at 3 weeks of age displayed a preference for fat over sugar in a macronutrient choice test, which was related to changes in dopamine-associated signaling pathways in the ventral striatum, including increased ΔFosB, cyclin-dependent kinase 5 (CDK5), and phospho-DARPP-32 Thr-75 (Teegarden et al., 2009). In another study, adolescent access to a 5% w/v sucrose solution led to sucrose anhedonia during adulthood, accompanied by reduced neurogenesis in the HPC, both of which were reversed by chronic antidepressant treatment (Gueye et al., 2018). In rats exposed to a high fat and high sucrose WD during early adolescence, blunted preference for sucrose and saccharin to coincide with reduced catechol-o-methyltransferase, an enzyme that can degrade dopamine, gene expression in the mPFC, a molecular feature which was absent when preference was restored at adulthood (Rabasa et al., 2016). Finally, female mice that had an anhedonic response to sucrose after having been exposed to a 60% kcals WD during adolescence exhibited increased dopamine transporter and reduced tyrosine hydroxylase expression in the VTA, reduced D1R and D2R expression in the ACB, and reduced D1R and D2R in the mPFC, all of which suggest reduced dopamine availability in these regions. However, a chronic 60% kcals fat WD during adolescence led to sucrose anhedonia and increased dopamine protein levels in the mPFC, which was later reversed after switching to a chow diet for 4 weeks (Carlin et al., 2016). Overall, it is clear that WD influences reward-mediated behavior in part by inducing long-lasting changes in dopamine signaling in the ACB, VTA, and PFC.

### Summary

**FIGURE 3:**
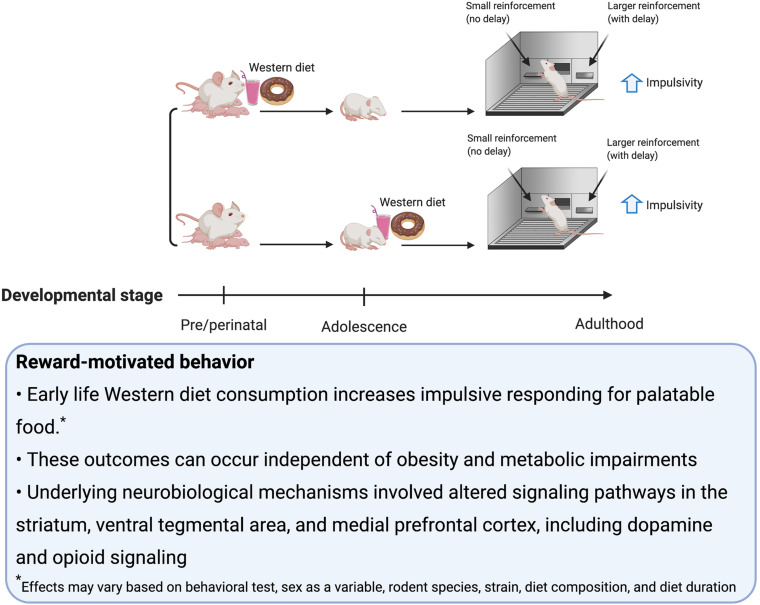
Summary of the effects of early life Western diet consumption on reward-motivated behavior.

Both perinatal and adolescent WD exposure in rodents has lasting effects on reward-motivated behavior in adulthood (Figure 3). Perinatal WD exposure has been shown to increase preference for drugs of abuse (Roversi et al., 2016; Sarker et al., 2019) and increase (Sarker et al., 2019) or decrease (Naef et al., 2008) sensitivity to drugs of abuse later in adulthood depending on the duration of perinatal WD exposure. Perinatal WD exposure is also associated with increased impulsivity (Grissom et al., 2015) and a decrease in behavioral flexibility (Wu et al., 2013). Furthermore, perinatal WD exposure in rats can lead to increased consumption of a WD later in life (Ong and Muhlhausler, 2014). Similar outcomes are associated with adolescent WD exposure in rodents. For example, sensitivity to the reinforcing effects of drugs of abuse is increased after WD exposure during adolescence and early adulthood (Blanco-Gandía et al., 2017) as well as locomotor sensitization to amphetamine (Naneix et al., 2017). WD consumption in adolescent rodents can alter macronutrient preference (e.g., carbohydrates vs. fat) depending on the type of macronutrient predominantly consumed during adolescence (Teegarden et al., 2009; Steele et al., 2019). However, increased preference does not necessarily lead to overconsumption as was seen in mice offered chronic WD access in adulthood (Teegarden et al., 2009). Findings from some studies also suggest that WD exposure from adolescence to adulthood can lead to anhedonia (Carlin et al., 2016; Naneix et al., 2016; Rabasa et al., 2016; Gueye et al., 2018). Additionally, adolescent WD consumption may reduce motivation for food rewards (Frazier et al., 2008; Vendruscolo et al., 2010; Privitera et al., 2011; Tantot et al., 2017; Gueye et al., 2018) and promote habitual as opposed to goal-directed behavior (Tantot et al., 2017). Finally, even in the absence of behavioral phenotype under basal conditions, adolescent consumption of a WD or sucrose solution during adolescence is linked to altered sensitivity to the effects of DA pharmacological agents on operant responding for food (Robertson and Rasmussen, 2017; Naneix et al., 2018) but not locomotor activity (Rabasa et al., 2016).

While perinatal and adolescent WD exposure can impact reward-motivated behavior, an obesogenic phenotype, as indicated by increased body weight and a perturbed metabolism, is not required for these effects to occur in rodents. Neural connections involving dopamine, opioid, and other neuropeptide systems between the VTA, mPFC, ACB, and various structures embedded within the brain reward circuitry regulate motivated behavior (Antonopoulos et al., 2002). Depending on the behavior observed, perinatal and adolescent WD exposure has been shown to either increase or decrease dopamine neurotransmission across regions of the reward circuitry by regulating dopamine uptake and metabolism, as well as the expression of dopaminergic, but also opioid and cannabinoid receptors. In summary, the altered reward-motivated behavioral effects seen with adolescent onset WD exposure is associated with various changes in DA transmission in reward circuitry, and can occur independent of WD effects on obesity and metabolic derangement.

## Social Behavior

### Behavioral Tests and Neural Substrates

In both humans and rodents, it is well known that dietary choices are influenced by social factors (Wouters et al., 2010; Salvy et al., 2012; Hsu et al., 2018; Pompili and Laghi, 2019). One mechanism by which social behavior can impact feeding behavior is by affecting reward systems. For example, in the case of “peer pressure,” being surrounded by peers has been shown to reduce inhibitory control by increasing reward sensitivity (Thiel et al., 2009; Logue et al., 2014). In addition, social isolation during adolescence can either increase or decrease sucrose drinking in rats during adulthood, suggesting that early life social isolation can change the incentive value of sucrose rewards (Van den Berg et al., 1999; Hong et al., 2012). However, while social behavior impacts dietary patterns and reward, the question as to whether dietary patterns impact social behavior remains largely underexplored. Given that the reward centers of the brain, such as the ACB, VTA, dorsal raphe nucleus, the lateral habenula, and mPFC are known to be activated by social reward (Chen and Hong, 2018) as well as food rewards (Kelley et al., 2005), it is reasonable to suspect that diet can impact social behavior and that this may be mediated by differences in oxytocin signaling, which modulates the neurons in the VTA and NAc to promote social reward (Dölen et al., 2013; Hung et al., 2017). In this section, we review the currently known effects of an obesogenic diet on social behavior, focusing on studies that examine rodent play behavior (three-chamber social interaction and social reciprocal interaction test), social memory (social-novelty preference test), and social avoidance (social defeat paradigm). We also discuss how diet may impact social behavior through the reward centers of the brain that are known to mediate the preference or reinforcement of social interactions.

### Prenatal/Perinatal WD Exposure

Prenatal and perinatal exposure to a WD has been shown to reduce social play behavior in rodents. For example, mouse dams maintained on a 60% kcal from fat WD diet had male and female offspring that displayed decreased sociability in the three-chamber social interaction test during young adulthood (Kang et al., 2014). Similarly, male rats born to dams that received a cafeteria diet of standard chow, milk chocolate, peanuts, and sweet biscuits during lactation spent less time socializing and had fewer social interactions in adulthood (Teixeira et al., 2020). Maternal consumption of a 60% kcal fat WD diet, initiated 8 weeks prior to conception, also reduced sociability and impaired social memory, deficits that were rescued by housing WD offspring with those from dams consuming a regular diet (Buffington et al., 2016). On the other hand, perinatal exposure to a 43% fat kcal WD diet had no impact on social behaviors during adulthood (Zieba et al., 2019), suggesting the % kcal from fat may be a key variable. Interestingly, WD exposure on the paternal side (pre-conception) also influences the social behavior of the offspring, as there were fewer play attack behaviors at PN 24–29 in both sexes and more defensive play behaviors in male rats if the rats were sired by males on a WD (60% kcals fat) for 60 days before mating (Korgan et al., 2018). In contrast, male and female rats that received a high-fat, high-sugar WD (60% kcals fat from solid diet, plus access to a 20% w/v sucrose solution) *in utero* up until PN 40 had increased play behavior (number of play attacks initiated) during adolescence (PN 37) compared to rats that received a control diet for the same duration (Hehar et al., 2016). One major difference between these studies is that the exposure to the diet was maintained in this study through the time of testing. Lastly, maternal WD feeding prevented the negative effect of early-life stress on social interactions in offspring (Rincel et al., 2016). Taken together, these studies suggest that prenatal/perinatal exposure to WDs may lead to reduced social behavior, though perhaps only when exposure to the diets are removed prior to testing, when diet fat content is high (∼60% kcal), and in the absence of early-life stress.

In each of the aforementioned studies, maternal/paternal obesity is a confounding factor, and thus it is difficult to determine whether observed effects are a result of the diet *per se*. For example, in the studies that found reduced social interactions as a result of a pre/perinatal WD, body weights (Kang et al., 2014; Korgan et al., 2018; Teixeira et al., 2020) and fat pads (Korgan et al., 2018; Teixeira et al., 2020) were increased in the offspring relative to controls. However, body weights and fat pads were also increased in the males that were provided the paternal WD and sired the offspring (Korgan et al., 2018), and increased weight was also seen in the dams provided with a maternal WD (Kang et al., 2014). Additionally, early life milk overnutrition starting at PN 2, induced by a reduction in litter size, results in overweight offspring and the males have a lower frequency of social play behavior (pouncing and pinning) without any differences in social behaviors unrelated to play (sniffing and grooming) (Carvalho et al., 2016). Such findings support the notion that weight gain alone may be sufficient to impact social behavior in male, but not female, rats. However, Buffington and colleagues (Buffington et al., 2016) observed profound social deficits in the absence of weight or adiposity differences in male offspring, even though WD fed dams had greater body weight. Taken together, these studies suggest that pre/perinatal exposure to WDs negatively impacts social behavior, and these effects are likely dependent on the duration of dietary exposure (whether the offspring are still on the diet at the time of testing) and the dietary fat content. Further studies are needed to determine the degree to which the effects of WD on social behavior are dependent on the development of obesity in either the parent or offspring.

The neural mechanisms by which pre/perinatal exposure to a WD impacts social behavior are thus far poorly understood. Among the reward system-related brain structures, the developing mPFC is a candidate mechanistic link as it is known to be involved in understanding social cues in rodent play behavior (Bicks et al., 2015). The HPC, which is known to be involved in social recognition (Uekita and Okanoya, 2011), may also be a neurobiological link between perinatal WD and altered social behavior. Supporting these notions, after a perinatal cafeteria diet, which reduced social interactions in adult offspring, levels of glutathione transferases, which are involved in cellular detoxification and oxidative stress, were greater in the mPFC (Teixeira et al., 2020). In the same study, levels of oxidative stress markers such as malondialdehyde (MDA) and superoxide dismutase (SOD) were higher in the HPC. Other potential mechanisms for reduced social behavior include increased whole brain neuroinflammation induced by IL-1β, TNFα and microglial activation, which was observed following *ad libitum* WD exposure 6 weeks prior to mating, during gestation, and during lactation (Kang et al., 2014). Importantly, both the reduced social behavior and elevated inflammatory marker expression across the brain normalized by switching to a healthy chow diet during lactation (Kang et al., 2014). Interestingly, social deficits in offspring of dams fed a WD were accompanied by dampened VTA LTP in DA neurons upon presentation of a novel mouse, as well as reduced oxytocin cell count in the hypothalamus and intestinal dysbiosis (Buffington et al., 2016). All of these impairments were restored by treatment with *Lactobacillus reuteri*, highlighting a role for gut flora on VTA function and associated social behaviors. Overall, while further research is required, these data suggest that early life WD exposure may impact neural processing in the mPFC, HPC, and VTA, and that a role for gut microbiota composition is an area for future investigation.

### Adolescent WD Exposure

Similar to perinatal exposure, consuming an obesogenic diet during adolescence impairs social memory and influences social interaction. More specifically, in adolescent male rats, either brief exposure to a WD (60% kcals fat) for 7–8 days starting at PN 27–28 or prolonged intermittent access to a WD (20% kcals fat, 39.6% kcals sucrose) diet for 2 h daily from PN 28–56 promoted deficits in social recognition memory, measured by the time spent exploring a novel rat vs. a familiar rat (Reichelt et al., 2019, 2020; Yaseen et al., 2019). Moreover, rats on an intermittent WD diet (2 hrs daily from PN 28–56) showed a reduction in the total duration of social contact when they were tested in a social interaction test with a novel rat (Reichelt et al., 2020). These findings were specific to conditions where the animal was allowed to interact with the rat prior to the WD feeding period on the day of the test. When tested after WD access, social investigation (sniffing, licking, grooming) was increased in rats fed WD. These data support that social interactions can be mediated by adolescent WD exposure, but it is important to note that only some social variables are affected, as the frequencies of social play behavior (pinning, pouncing) and of aggressive-like behavior (biting, boxing, overt physical harm) were not affected by the dietary exposure (Reichelt et al., 2020). Additional work revealed that intermittent access to a WD during adolescence impaired both social interaction and social memory, but had no impact on social odor preference as all rats preferred bedding soiled by a conspecific (Reichelt et al., 2020). Adolescent consumption of a 45% fat diet, but not 38% fat and 20% fructose diet, also reduced social interaction during adulthood (Gancheva et al., 2017), suggesting a predominant role for dietary lipids. Consistent with this framework, free access to a 5% sucrose solution during adolescence had no effect on social behaviors during adulthood (Gueye et al., 2018), while consuming a 60% fat diet for 7 weeks reduced social interactions in both the 3-chambers test and the social reciprocal interactions test, in addition to reducing ultrasonic vocalization upon female encounter (Yang et al., 2020). Conversely, male mice on a WD (45% kcal from fat) or cafeteria diet from PN 28–49 before switching to standard chow did not display differences in social behavior in the three-chamber social interaction test in adulthood at PN 73+ relative to controls (Fülling et al., 2020), suggesting that it is possible for deficits in social behavior to be reversed by a dietary intervention. Thus, additional studies to determine the duration of exposure to WDs necessary to impact social behavior, as well as whether or not the effects are reversible with dietary intervention are warranted.

Although adolescent WDs lead to impaired social memory and differences in social interactions, WDs can also protect against social defeat stress. For example, male rats that received 4-h daily access to a 45% kcals fat WD daily for 9 weeks after weaning were more resilient to social defeat stress in adulthood (PN 63), as measured by resident-intruder interactions (spending less time in submissive postures and having a longer latency to submit to the resident) (MacKay et al., 2017). Resiliency to social defeat stress was also demonstrated by another study that found that consuming a 45% kcals fat WD *ad libitum* from weaning onwards in male mice did not result in social avoidance behavior in adulthood when tested using a social interaction test after social defeat stress (Finger et al., 2011). Together, these studies show a protective effect of adolescent WD on social defeat stress such that a WD can be anxiolytic under stressful circumstances, a notion supported by the fact that the rodents in both of the aforementioned studies displayed reduced anxiety with WD exposure after social defeat stress as measured by open field (MacKay et al., 2017) and the light-dark box test (Finger et al., 2011).

While some of the animals that showed deficits in social recognition memory after having consumed a WD during adolescence did not show differences in body weight (Reichelt et al., 2019, 2020; Yaseen et al., 2019), fasting glucose, or cholesterol (Yaseen et al., 2019), those that were impaired in social memory did have higher energy intake and fat mass (Gancheva et al., 2017; Reichelt et al., 2019, 2020; Yang et al., 2020) and increased systemic levels of leptin (Yaseen et al., 2019). However, the rodents resilient to social defeat stress after WD exposure either showed increased weight gain with increased plasma leptin and insulin levels (Finger et al., 2011) or no differences in body weights, body fat, glucose tolerance, caloric intake, or basal corticosterone levels compared to controls (MacKay et al., 2017). In cases where no differences in social interactions were found, mice fed a 45% kcals fat WD but not cafeteria diet had increased body weight and energy intake relative to controls (Fülling et al., 2020), suggesting that obesogenic phenotypes after adolescent WD exposure likely do not influence social behavior.

Similar to the consequences of perinatal WDs on social behavior, adolescent WDs may affect social behavior by impacting the PFC. For instance, social memory deficits were associated with impaired long-term potentiation in the mPFC and reduced protein levels of oxytocin in mPFC tissue. These deficits were reversed by administration of oxytocin systemically or the selective oxytocin receptor agonist, [Thr4,Gly7]-oxytocin in the mPFC (Yaseen et al., 2019), which supports the idea that intact oxytocin signaling is necessary for rodents to recognize a familiar conspecific animal (Ferguson et al., 2000). Furthermore, the effects on social behavior and memory were associated with reduced expression of monoamine oxydase and COMT genes in the mPFC (Reichelt et al., 2020), suggesting that monoamine activity possibly related to dopamine was altered by WDs. In another study, the same group reported similar molecular outcomes in the mPFC, as well as reduced hippocampal monoamine oxydase and changes in gut microbiota (Reichelt et al., 2020). Impairments in social memory were further determined to be associated with reduced parvalbumin interneurons in the mPFC and increased co-expression of perineuronal nets on parvalbumin interneurons, both of which are involved in neuroplasticity (Reichelt et al., 2019). Male mice showing alterations in social behaviors also displayed blunted neuronal activity in the mPFC upon presentation of a social stimulus, and mPFC expression of senescence-related genes was upregulated, especially in astrocytes and microglia (Yang et al., 2020). Finally, given increased serum levels of both triglycerides and reactive species, it has been hypothesized that altered lipid peroxidation mediates the social impairments associated to adolescent WD feeding (Gancheva et al., 2017). Overall, these findings suggest that adolescent WD exposure may impact social behavior through oxytocin signaling, monoamine enzymes, and changes in mPFC neuroplasticity.

### Summary

**FIGURE 4:**
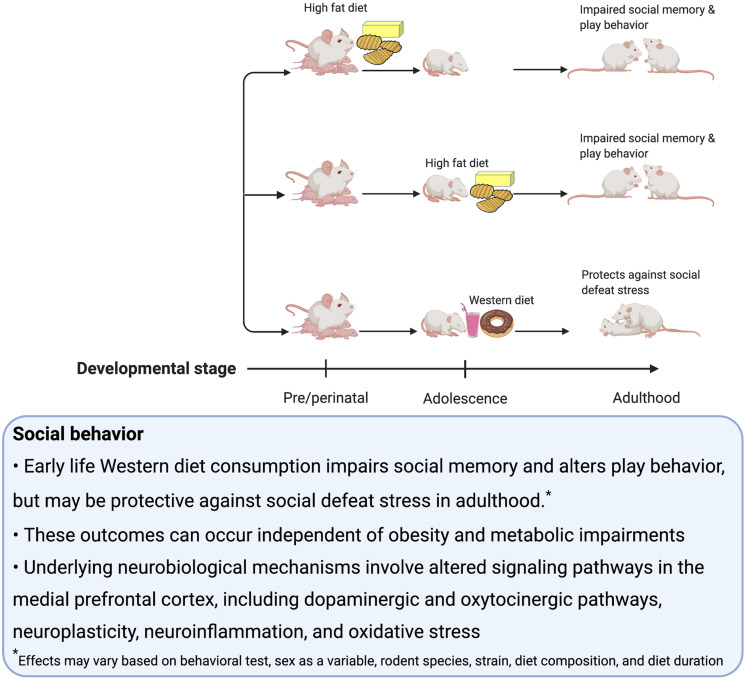
Summary of the effects of early life Western diet consumption on social behavior.

The studies reviewed here suggest that consumption of a WD during early life periods of development impair social-based memory and alters play behavior (e.g., reduces play attacks), but also that these same diet exposures may be protective against social defeat stress (Figure 4). These effects are likely independent of an obesogenic phenotype and may include a mechanism involving mPFC dopaminergic and oxytocinergic pathways as well as changes in neuroplasticity, neuroinflammation, and oxidative stress. Given that the majority of these studies in this section only observed male rodents, further research investigating how early life WD consumption impacts social behavior in females is necessary in the future.

## Conclusion

In this review, we present behavioral and neurobiological evidence from preclinical rodent models highlighting that consumption of (or perinatal exposure to) a WD during critical periods of development can lead to neurocognitive dysfunction later in adulthood. In particular, we highlight the detrimental effects of WDs high in fat (particularly saturated), sugar, or a combination of the two on the following cognitive domains: anxiety-like behavior, learning and memory function, reward-motivated behavior, and social behavior (Supplementary Table 1).

During the perinatal period, exposure to WDs results in impairments in each of these cognitive domains in rodents when tested during adolescence and/or adulthood. For example, increased anxiety-like behavior is associated with prenatal/perinatal WD exposure unless the rodents were also subjected to early life stress, where prenatal/perinatal WDs ameliorates anxiety-like behavior. Additionally, WD exposure during the perinatal period impairs episodic memory and conditioned odor aversion learning and extinction but has no effect on contextual or cued fear conditioning. Furthermore, effects on reward-motivated behavior are also common after prenatal/perinatal WD exposure, including addictive-like behaviors to drugs of abuse, increased impulsivity, reduced behavioral flexibility, and reduced motivation to work for food reward despite increased consumption when the rewarding food is made freely available. Finally, prenatal/perinatal WD exposure reduces social memory and prosocial behavior in rodents. Importantly, these effects are often seen despite the progeny being maintained on a standard, low-fat chow after weaning. Further research is necessary to evaluate whether behavioral effects are specific to a certain stage within the prenatal/perinatal period, as some studies found certain effects were specific to late gestation or lactation.

Long-term access to WDs during adolescence increases anxiety-like behavior in adulthood, although this effect may not be long-lasting given that anxiety-like behavior is not seen in rodents that had the WD removed for an extended period of time. Similar to what occurs after prenatal/perinatal WD exposure, adolescent WD exposure is protective against the development of anxiety-like behavior when consumed during periods of early life stress. Both acute and chronic consumption of WDs has deleterious effects on learning and memory processes, including impaired spatial and episodic memory, cued fear learning and extinction, aversive memory, and reversal learning. With regards to reward-motivated behavior, WD exposure during adolescence shifts dietary preferences in adulthood towards a diet similar to what the animal was exposed to during adolescence. Additionally, adolescent WD exposure increases impulsive responding for food rewards, reduces motivation for food rewards, and increases drug-seeking behavior. WD consumption during adolescence also impairs social memory and affects social interaction behavior in adulthood, yet reduces stress induced by social defeat. Altogether, the literature indicates that adolescent exposure to WDs promotes enduring cognitive impairments but may also mitigate stress responses. More research is necessary to determine whether similar effects are observed in female rodents, and whether sex hormones are a critical variable.

In many instances, behavioral deficits associated with early life WD exposure occurred independent of obesity-associated outcomes such as increased body weights, adiposity measures, hyperphagia, or negative metabolic outcomes. Further, in many cases the neural substrates associated with each cognitive domain are impacted by WD exposure during development, such as increased oxidative stress, inflammation and microglial activation, as well as altered neurotransmission, neurotrophic signaling, synaptic plasticity, and reduced neurogenesis. What remains to be understood is whether or not these cognitive deficits can be reversed given some type of intervention (e.g., dietary, exercise, microbiome) and whether or not these cognitive consequences have transgenerational effects.

Evidence reviewed herein establishes that WD exposure in rodents during the perinatal and/or adolescent period promotes long-term deficits in anxiety-like behavior, learning and memory, reward-mediated behavior. While this review focused on rodent studies, similar changes in behavior in humans have been reported, where healthier diets are positively associated with better cognitive outcomes (Baym et al., 2014; Zahedi et al., 2014; Tandon et al., 2016; Borge et al., 2017; Cohen et al., 2018). The rodent literature reviewed here adds important insight into the mechanisms underlying poor cognitive outcomes following early life WD consumption and may help guide translational research and dietary recommendations in humans.

## Author Contributions

All authors contributed to the idea for the manuscript. LT and LD-S wrote and edited the manuscript. SK and EN edited the manuscript, provided vital input to shape the manuscript, and contributed to the writing. All authors approved the final manuscript. The figures were created using BioRender.com by LT, and reviewed by all authors.

## Conflict of Interest

The authors declare that the research was conducted in the absence of any commercial or financial relationships that could be construed as a potential conflict of interest.
